# Recent Advances in Functional Polymers Containing Coumarin Chromophores

**DOI:** 10.3390/polym13010056

**Published:** 2020-12-25

**Authors:** Ines Cazin, Elisabeth Rossegger, Gema Guedes de la Cruz, Thomas Griesser, Sandra Schlögl

**Affiliations:** 1 Polymer Competence Center Leoben GmbH, Roseggerstrasse 12, 8700 Leoben, Austria; ines.cazin@pccl.at (I.C.); elisabeth.rossegger@pccl.at (E.R.); 2 Department Polymer Engineering and Science, Institute Chemistry of Polymeric Materials, Montanuniversitaet Leoben, Otto Glöckel-Strasse 2, 8700 Leoben, Austria; gema-del-carmen.guedes-de-la-cruz@unileoben.ac.at (G.G.d.l.C.); thomas.griesser@unileoben.ac.at (T.G.)

**Keywords:** coumarin, functional polymers, self-healing, biomedical applications, shape-memory

## Abstract

Natural and synthetic coumarin derivatives have gained increased attention in the design of functional polymers and polymer networks due to their unique optical, biological, and photochemical properties. This review provides a comprehensive overview over recent developments in macromolecular architecture and mainly covers examples from the literature published from 2004 to 2020. Along with a discussion on coumarin and its photochemical properties, we focus on polymers containing coumarin as a nonreactive moiety as well as polymer systems exploiting the dimerization and/or reversible nature of the [2πs + 2πs] cycloaddition reaction. Coumarin moieties undergo a reversible [2πs + 2πs] cycloaddition reaction upon irradiation with specific wavelengths in the UV region, which is applied to impart intrinsic healability, shape-memory, and reversible properties into polymers. In addition, coumarin chromophores are able to dimerize under the exposure to direct sunlight, which is a promising route for the synthesis and cross-linking of polymer systems under “green” and environment-friendly conditions. Along with the chemistry and design of coumarin functional polymers, we highlight various future application fields of coumarin containing polymers involving tissue engineering, drug delivery systems, soft robotics, or 4D printing applications.

## 1. Introduction

**Figure 1:**
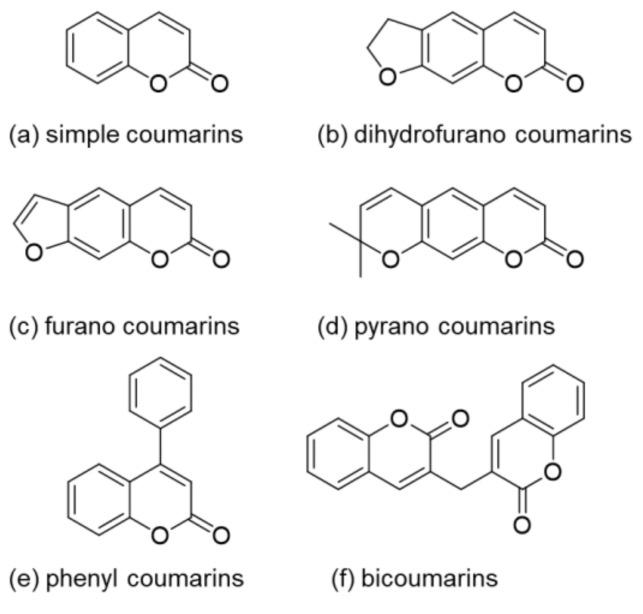
Structures of the different coumarin classes.

Coumarin (2H-1-benzopyran-2-one) is an oxygen containing heterocycle and belongs to the subcategory of lactones. It is named after the French word for tonka bean *(Dipteryx odorata) Coumarou* as Vogel first extracted coumarin from tonka beans in 1820 [1]. Later, coumarin was also isolated from sweet clover, bison grass, and woodruff [2]. There exist six different basic natural types of coumarin: (a) simple coumarin derivatives, (b) dihydrofurano coumarin derivatives, (c) furano coumarin derivatives, (d) pyrano coumarin derivatives (linear and angular), (e) phenyl coumarin derivatives, and (f) bicoumarin derivatives (Figure 1) [3].

As they are secondary metabolites of bacteria, plants, and fungi [3,4], coumarins appear in many different natural sources such as essential oils, fruits, green tea, and other foods [3,5]. Although these natural compounds occur in various parts of different plants, their highest concentration can be found in fruits, roots, stems, and leaves. However, their concentration distribution is influenced by environmental and seasonal changes [6]. Apart from plants, microorganisms are also a natural source of coumarins. For instance, novobiocin and coumermycin have been extracted from *Streptomyces* and aflatoxins from *Aspergillus* species [7,8]. While aflatoxins are very toxic fungal metabolites [6], novobiocin and coumermycin are members of an antibiotics group as they are able to inhibit DNA gyrase. All members of this antibiotics group feature a 3-amino-4-hydroxy-coumarin moiety and a substituted deoxysugar [9].

The isolated coumarins are mostly biologically active and show antimicrobial, antibacterial, antifungal [3,10,11,12,13,14,15,16,17,18,19], and antiviral activity [17,18]. Others reveal antioxidant [17,20,21,22], anti-inflammatory [3,22], and/or anticorrosive [23] activity. There are also reports about the usage of coumarin derivatives for medical application. For example, some types of coumarin are used for Alzheimer disease treatment due to their ability to inhibit acetylcholinesterase (AchE) [17,18,24]. Moreover, authors reported anti-HIV [25,26], anticancer [5,17,18], and anticoagulant [3,4] activity.

**Figure 2:**
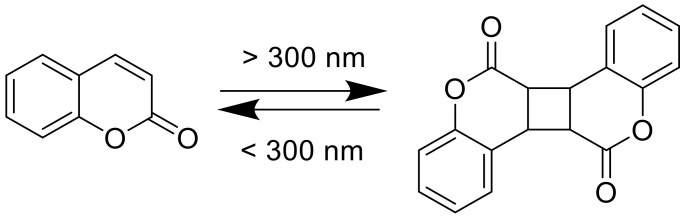
Photodimerization of coumarin.

Coumarins are not only interesting because of their bioactivity but also due to their photoreactivity. In 1902, Ciamician and Silber investigated the photodimerization of coumarin under UV light exposure (>300 nm), in ethanol, or in aqueous solutions (Figure 2) [27].

During photoirradiation, four different types of dimers are being formed: *anti* head-to-head, *anti* head-to-tail, *syn* head-to-head, and *syn* head-to-tail. Krauch et al. investigated the photocleavage of *anti*-head-to-head dimers in dioxane with wavelength <310 nm, in 1966 (Figure 2) [28]. Additionally, coumarin and its derivatives are highly fluorescent in the visible light range. Researchers started their studies on photophysics of coumarin moieties in the 1940s. In the late 1950s, Wheelock and his coworkers showed a shift of the fluorescence band by substitutions on the coumarin structure [29,30]. These properties enable the use of coumarin molecules in numerous different application fields such in organic light emitting diodes (OLEDs) [31,32,33,34], optical data storages [35,36], laser dyes [37,38], or drug delivery systems [39,40,41,42,43,44].

Researchers from different areas, such as medicine, polymer science, or biology, are working on coumarins due to their versatile properties [29]. However, the isolation from natural resources is time consuming and not profitable. Therefore, the research regarding the synthesis of coumarin and its derivatives has gained increased attention. Lončarić et al. published a review of numerous synthesis strategies in 2020 involving Perkin reaction, Knoevenagel condensation, Pechmann condensation, Wittig reaction, Baylis-Hillman reaction, Claisen rearrangement, and Vilsmeier–Haack or Suzuki cross-coupling reaction, to name only a few examples [18].

Trenor et al. reviewed the use of coumarin moieties in the design of functional polymers in 2004. They focused on coumarin-containing polymers in electro-optical studies, the development of photoreversible systems, coumarin in biopolymers, polymerizations, chiral stationary phases for HPLC, and fluorescent tags and fluoroprobes [29]. Since 2004, there has been a steadily growing interest in using the versatility of coumarin chromophores in the design of functional polymers. This current contribution serves as an update to the previous review from Trenor et al. and will focus on the latest developments in coumarin functional polymers. In particular, this review will mainly cover studies published from 2004 to 2020 and will give a deep insight into the different reaction mechanisms of coumarin, the synthesis routes for coumarin-functionalized polymers, the applications of polymers with coumarin as nonreactive moiety and the advanced application fields of polymers with coumarin as reactive moiety. Self-healing, shape-memory polymers, drug delivery systems, soft robotics, and 4D printing are only a few examples of the broad new application field.

## 2. Photoreaction Mechanisms of Coumarin and Its Derivatives

Due to the excellent electronic, photophysical, and photochemical properties of coumarin and its derivatives, these chromophores can undergo various photoreactions and play a key role in different fields of applications. Prominent examples are photoactive surfaces relying on photofuses, controlled release of biochemical substances based on caged compounds, introduction of (reversibly) cross-linkable moieties in polymers, and generation of reactive chemical species by exploiting coumarin compounds as photoinitiators. Based on the relevant applications, this chapter has been divided into three parts in accordance with its reactivity and mechanisms involved.

### 2.1. Photocleavage of Coumarin-Caged Compounds and Photolabile Surfaces Bearing Functional Coumarin Groups (Photofuses)

**Figure 3:**
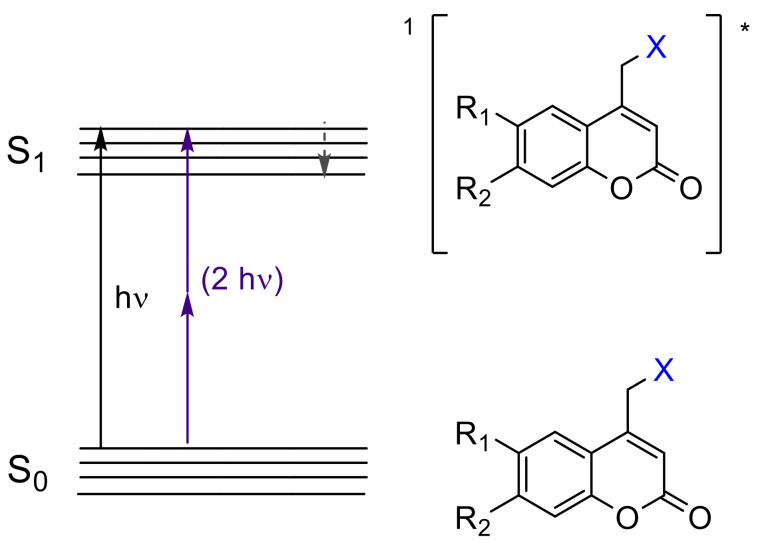
Excited singlet state (S_1_)* of coumarin-4-yl derivatives by one-photon (UV light) and two-photon transfer (visible and near-IR light). The asterisk (*) denotes the excited state of the molecules.

The irreversible response of (coumarin-4-yl)methyl derivatives upon light exposure has been particularly exploited as a tool for temporally and spatially controlled probes of cell-based processes [45,46] as well as for the generation of patterned surfaces in different field of applications including bioanalytical science, cell biology, tissue engineering, etc. [47,48,49,50,51]. Moreover, its ability to undergo photolysis by nonresonant two-photon excitation (Figure 3), at high light intensity, facilitates salient properties of this chromophore such as: (a) “phototherapeutic window” between 650 and 950 nm with lower scattering and reduced phototoxic effect [46] and (b) orthogonal protecting group based on wavelength-selective response [51].

**Figure 4:**
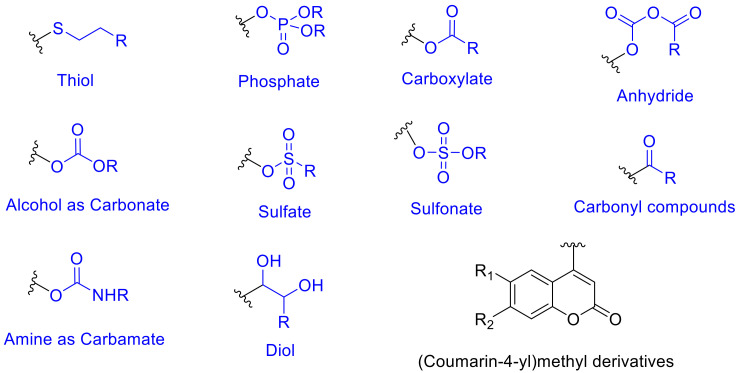
Coumarin-4-yl derivatives employed as caged compounds and photofuses.

**Figure 5:**
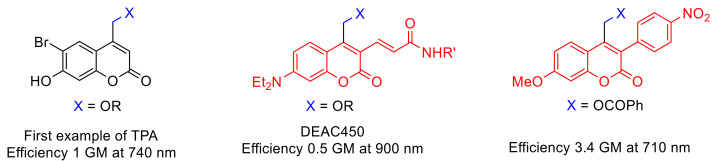
Coumarin-caged compounds undergoing two-photon absorption (TPA).

The caged compounds and photofuses are constituted from a wide range of functional groups: thiol, phosphate, carboxylate, anhydride, sulfate, alcohol as carbonate, amine as carbamate, etc. [46,52] (Figure 4). Recently, the scope of these derivatives has grown, improving their long-wavelength absorption and uncaging efficiency (GM) by extending π-conjugation at 3-position on the coumarin ring [53,54,55,56] (Figure 5).

**Figure 6:**
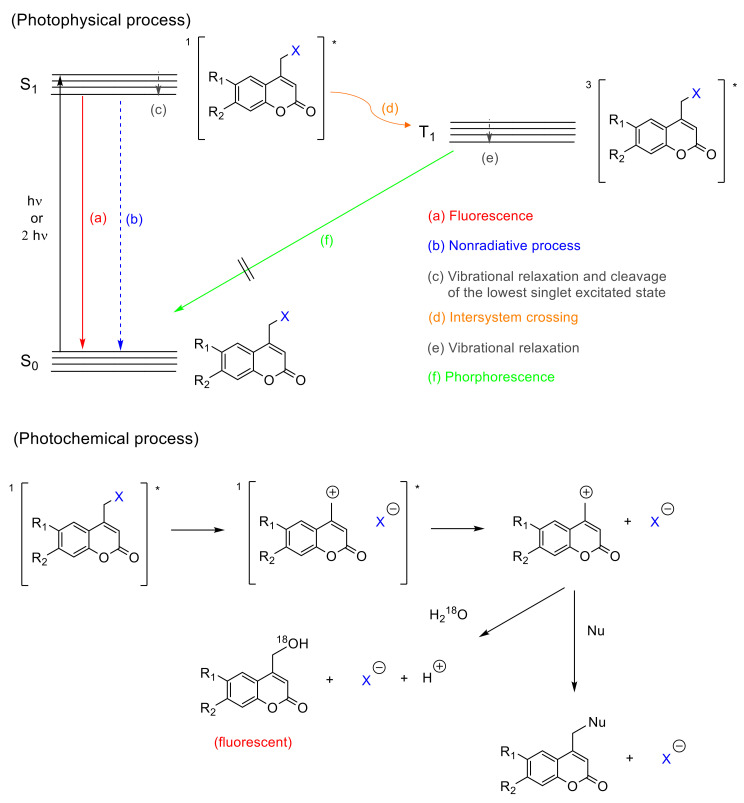
Mechanism of the photocleavage reaction of coumarin derivatives. The asterisk (*) denotes the excited state of the molecules.

From the mechanistic point of view, after one-photon absorption (UV light) or two-photon absorption (visible or near-IR light), the relaxation to the lowest level of the excited singlet state (S_1_) takes place [52] (^1^[Coum-X]* in Figure 6). This intermediate might progress in one of three ways: (a) fluorescence, (b) nonradiative process, and (c) cleavage of the C-X bond forming a singlet ion pair (Figure 6a–c). The factor that determines the subsequent path is the reaction rate constant (*k*). When *k_f_*(fluorescence rate constant ~10^8^ s^−1^) [52] and *k_nr_* (nonradiative rate constant ~10^8^ s^−1^) [52] are smaller than *k_cl_*(cleavage rate constant ~10^9^ s^−1^) [52], the photolysis predominates. In this case, any possible photophysical deactivation (a) or (b) is hampered due to the higher stability of the excited state and its subsequent separated species. These pathways have been well studied by Bendig et al. in ester and amide derivatives [52,57,58,59,60].

There is evidence that the cleavage of the C-X bond in coumarin derivatives follows a heterolytic route. A study carried out with ^18^O-labeled water, also by Bendig et al., pointed the generation of ionic species by S_N_1 mechanism, after isolating 4-(^18^O-hydroxymethyl)coumarin out. This mechanism is evidenced by the higher efficiencies obtained using polar protic solvents and good leaving groups (X with low pk_a_ values) in the coumarin derivatives [58] (solvent-assisted photoheterolysis, Figure 6). Additionally, 4-methylcoumarin is only detected in trace amounts, which is expected to be the main product in a homolytic cleavage and favors the heterolytic hypothesis [58]. However, the possibility of a homolytic route should not be entirely excluded, since a singlet electron transfer from the homolytic bond cleavage could drive to the ion pair as well [58].

There is also a controversy regarding a possible intersystem crossing (Figure 6d). Studies by Arai et al., carried out with 7-aminocoumarins, showed a weak transient band in the range of 500–700 nm (Figure 6e) that was ascribed to the triplet state. This result suggests that the photolysis of caged compounds bearing this chromophore partially proceeds from the triplet excited state, which is convenient since the deactivation by recombination of the separated species is not allowed from a quantum mechanics point of view [61]. On the contrary, Bendig et al. postulated that this process proceeds only from the excited singlet state, based on the lack of phosphorescence (Figure 6f) characteristic for triplet states, and the trace amount of 4-methylcoumarin, which should be formed from the radical species generated in the triplet state after the intersystem crossing (Figure 6d) [58].

Once the singlet ion pair is formed after photolysis, according to Bendig et al., the ions can escape from the solvent cage and the (coumarin)methylium cation reacts with any nucleophile found in the medium.

### 2.2. [2πs + 2πs] Photocycloaddition Reaction of Coumarin Groups

The [2πs + 2πs] photocycloaddition of coumarin derivatives upon irradiation with UV light (>300 nm), to generate cyclobutane derivatives with itself or even with another double bond in the reaction medium, has been widely employed in the synthesis of polymeric networks [41,62,63,64,65,66,67,68]. The advantages of this method are the creation of a network without extra monomers or photoinitiators [66] and the possibility to reverse the process (by regenerating the double bonds using radiation wavelength <290 nm). This reversible process provides networks with diverse properties such as self-healing [68,69,70,71], reversible wettability [72], reversible thickness [73], and reversible assembly in supramolecular architectures [74,75], which makes the photodimerization of coumarin a useful reaction pathway in the field of polymers.

**Figure 7:**
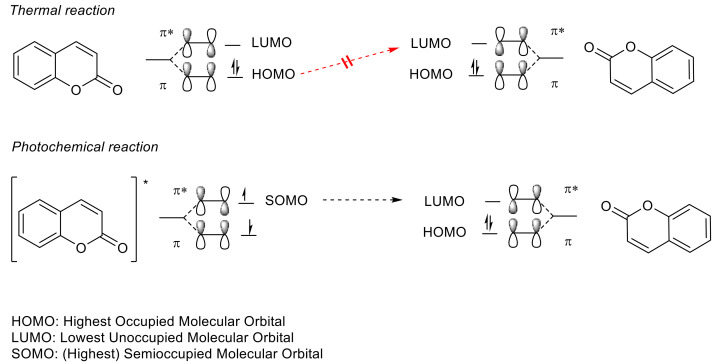
Conservation of orbital symmetry for [2πs + 2πs] cycloaddition of coumarin. The asterisk (*) denotes the excited state of the molecules.

**Figure 8:**
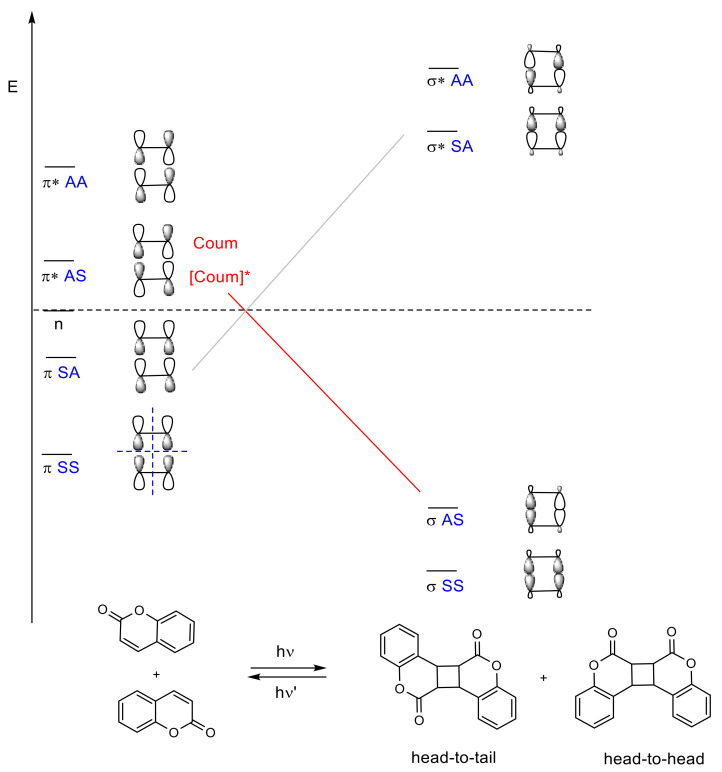
Energy and correlation diagram of the [2πs + 2πs] photodimerization of coumarin. The asterisk (*) denotes the excited state of the molecules.

Apart from a few exceptions [76,77], thermal [2πs + 2πs] cycloaddition processes are forbidden due to conservation of orbital symmetry [77,78]. Therefore, the photo-induced reaction (exposure with wavelengths in the UVA spectral region) takes place between the excited antibonding orbital π of one double bond (excited HOMO = SOMO = π*) and the antibonding orbital π (LUMO = π*) of another double bond (Figure 7). This way, the cyclobutane ring/the two new sigma bonds is/are formed from two π bonds (Figure 8).

**Figure 9:**
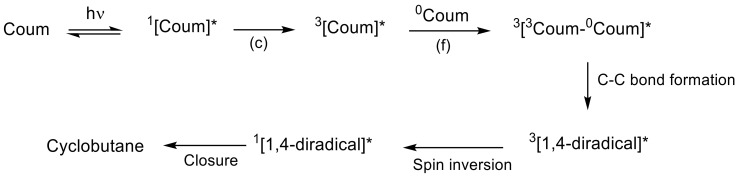
Possible mechanism of cyclobutane formation from [2πs + 2πs] photodimerization of coumarin. The asterisk (*) denotes the excited state of the molecules.

The mechanistic approach of the [2πs + 2πs] photocycloaddition of coumarin can be rationalized by comparing the hypothetical mechanism between enones and alkenes [79], since coumarin derivatives are formally enones. After one- or two-photon absorption of the ground state of coumarin (^0^Coum) [80], the excited singlet state ^1^[Coum]* is produced by either *n* → π* or π → π* transition (Figure 9) [77,78]. This intermediate might progress in one of the following four pathways: (a) fluorescence, (b) nonradiative process, (c) intersystem crossing to an excited triplet state ^3^[Coum]*, and (d) the formation of a singlet exciplex ^1^[^1^Coum-^0^Coum]* (Figure 6). Since the intersystem crossing in six-membered cyclic enones is an efficient process, path (c) is most common [77,78,81].

In turn, the triplet state ^3^[Coum]* can either (c) decay back to the ground state or (f) combine with the ground state of coumarin (^0^Coum) to generate a triplet exciplex ^3^[^3^Coum-^0^Coum]*. The exciplex can form a carbon–carbon bond and produce a triplet 1,4-diradical, ^3^[1,4-diradical]*, which must undergo spin inversion to the singlet diradical, ^1^[1,4-diradical]*, before a closure to the cyclobutane ring occurs (Figure 9).

In a more recent work, Bach et al. studied the role of a Lewis acid in the intramolecular [2πs + 2πs] photocycloaddition of coumarin derivatives and dihydropyridones [82]. In this study, it was pointed out that the uncatalyzed [2πs + 2πs] photocycloaddition of coumarin derivatives goes through its singlet state, while the use of a Lewis acid seems to stabilize the singlet state and facilitates the intersystem crossing (ISC), and therefore, the coumarin photocycloaddition in this case occurs via the triple state.

In [2πs + 2πs] photocycloadditions, four possible products could be obtained: *syn* head-to-head, *syn* head-to-tail, *anti* head-to-head, and *anti* head-to-tail (Figure 8). Their stereoselectivity and regioselectivity depends on several factors (polarity of solvents, addition of a sensitizer, crystal packing, and distances between double bonds) and is related to the reaction medium (in solution, in solid, or even in inclusion complex). A review published by Trenor et al. details the effects of radiation doses, solvent, and concentration over the [2πs + 2πs] photocycloadditions in coumarin derivatives in different media [29].

Reversible [2πs + 2πs] photocycloaddition, leading to the cleavage of the cyclobutane rings, can be symmetric (the same double bonds are generated) or asymmetric (two different types of doubles bonds). The photocleavage of cyclobutane coumarin dimers was studied by Hasegawa et al., concluding that the photocleavage, in this case, is symmetric [29] since the cleavage of cyclobutanes attached to five- or six-member ring takes place and maintains the more stable ring. On the other hand, Görner et al. proposed, on the basis of their studies in presence and absence of triplet state sensitizers, that the photocleavage occurs via a nonfluorescent and short-lived singlet state [83]. In addition, in turn, Motzkus et al. corroborated Görner’s results and proposed a ring scission by steps with the formation of intermediates [84].

Joy et al., reported two different mechanisms for coumarin chromophores in photoresponsive and biodegradable functional polyesters [85]. In this case, irradiation (350 nm) of the coumarin chromophores bearing good leaving groups (ester or phosphate group) in the 4-methyl position leads to the cross-linked network, while the scission of the coumarin photodimers is obtained upon irradiation at 254 nm.

### 2.3. Coumarin Derivatives Serving as Photoinitiators

In pursuit of photopolymerization reactions [86] involving novel photoinitiators (PIs), which possess strong two-photon absorption (TPA) in the visible/near-IR region [87,88], and with the aim of providing better features for 3D printing [87,88,89], three-dimensional optical data storage [90,91], and microfabrication [90,92,93], research into coumarin and its derivatives has been growing up to now. These applications, which use TPA, benefit from the localized excitation of the photocurable resin near to the focal volume of the laser, due to its probability of being proportional to the square of light intensity [90,93].

Different types of coumarin-based two-photon photoinitiators (2PIs) have been developed: (a) *unimolecular,* (b) *bimolecular,* and (c) *multicomponent systems* [88].

**Figure 10:**
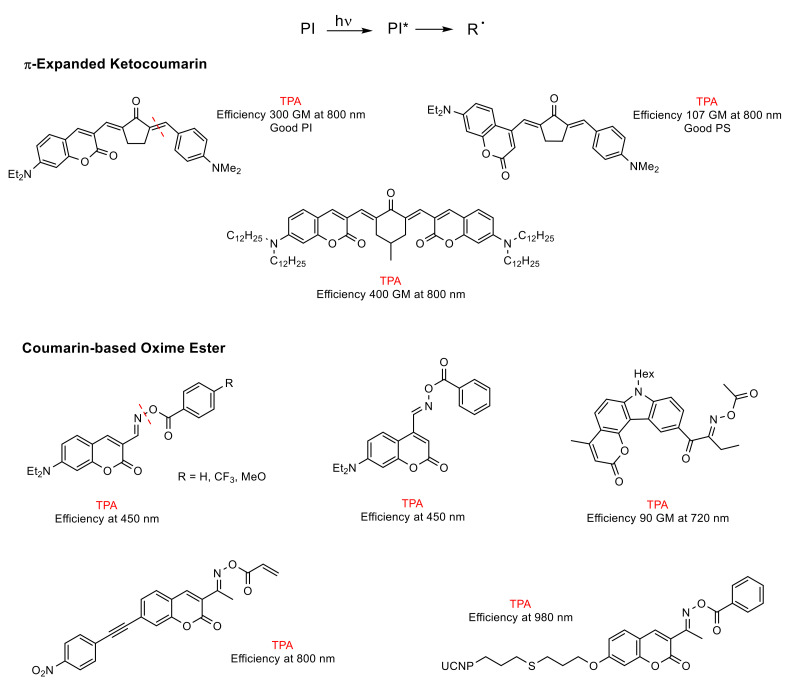
Unimolecular coumarin-based photoinitiators (PIs). The asterisk (*) denotes the excited state of the molecules.

**Figure 11:**
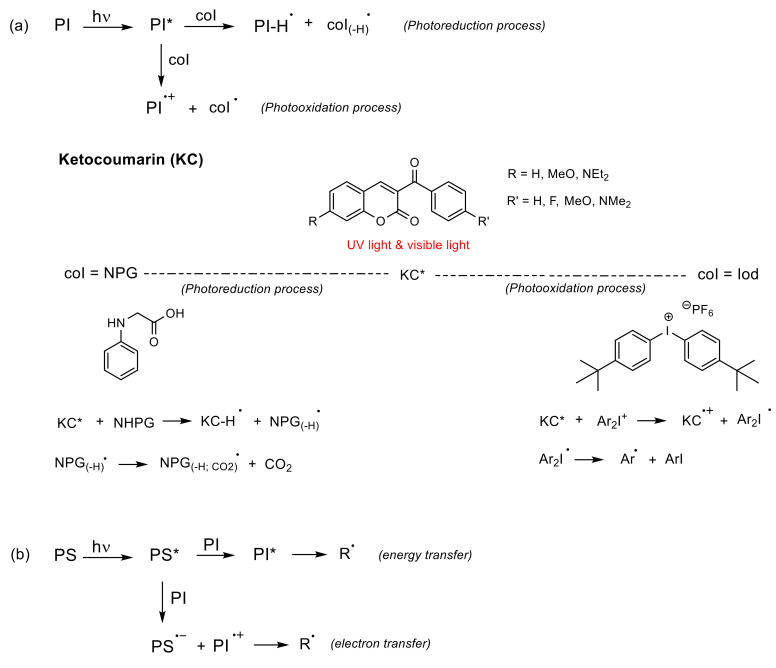
Bimolecular coumarin-based PIs. (**a**) Photoreduction process where the coI transfers an electron and a proton into the excited state of the PI, and photooxidation process where the excited state of PI transfers an electron into the coI; (**b**) The excited PS might transfer energy or an electron into the PI. The asterisk (*) denotes the excited state of the molecules.

(a) *Unimolecular system* (photocleavable PI, Type I): upon absorption, the excited state of the PI undergoes a homolytic cleavage to produce free radicals. Subsequently, an electron transfer from one of these radicals to a monomer generates the radical anion species responsible for the polymerization [86,90]. In contrast to the photocleavage mechanism in (coumarin-4-yl)methyl derivatives, the radical species are generated from the triplet state after the intersystem crossing [87,89,92,94]. This type of coumarin-based 2PIs is generally constituted by conjugated carbonyl groups (photocleavage of a double bond in α-carbonyl position) [92,94] or by oxime-ester (photocleavage of an N-O bond) [87,95,96,97] (Figure 10).
(b) *Bimolecular system* (PI/coI (co-initiator) or PI/PS (photosensitizer), Type II) [86]: Once the PI is excited in the PI/coI system, a transfer of an electron/proton takes place between both compounds (see Figure 11a), thus resulting in radicals or ions that initiate the polymerization reaction. Some examples of typical coIs, employed in combination with ketocoumarins as PIs, are *bis-*(4-*tert*-butylphenyl)iodonium hexafluorophosphate (Iod or SpeedCure 938), *N*-phenylglycine (NPG), and ethyl 4-(dimethylamino)benzoate (EDB) [89]. The triplet state pathway is also possible in the mechanism of bimolecular system (KC/Iod), since free energy change for an electron transfer (∆G_et_) from the aforementioned state is favorable [88,89].

In contrast, in the PI/PS system, a transfer of energy or an electron occurs from the excited PS to the PI after irradiation, thereby generating radical or ions [86], as shown in Figure 11b. To the best of our knowledge, there are cases of coumarin derivatives used as photosensitizers, albeit only in multicomponent systems [98].

(c) *Multicomponent system* (three or more compounds): this system involves combinations that allow an improvement in the performance of PIs under the conditions required by the applications [86]. Thus, its mechanism is rather complex.

**Figure 12:**
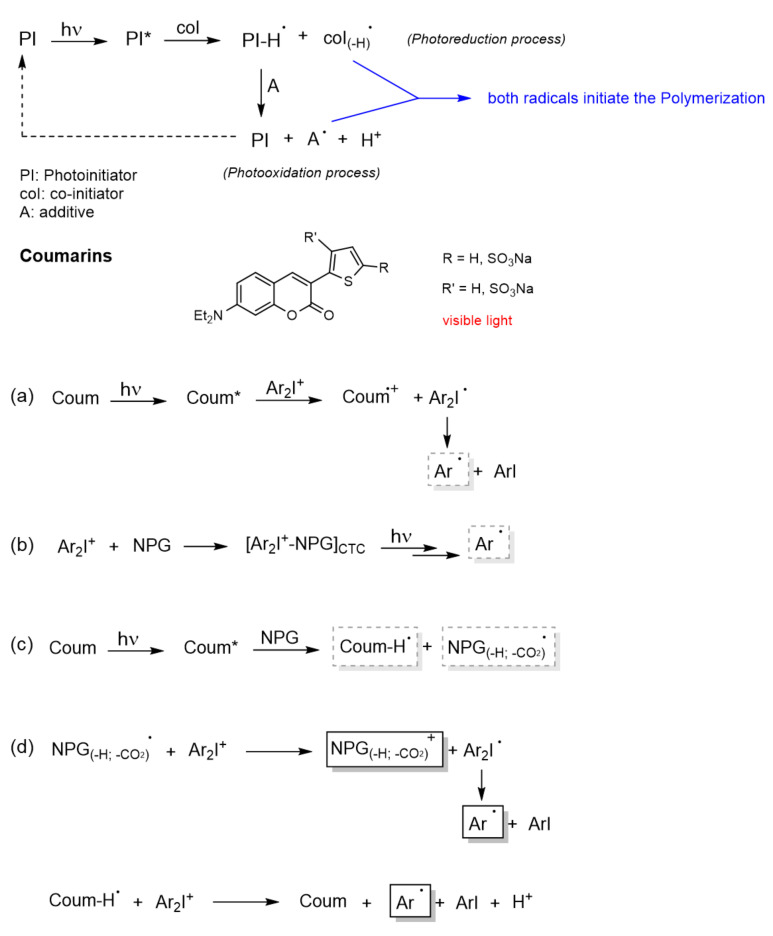
Multicomponent coumarin-based PIs. Mechanistic pathway of the systems: (**a**) Coum/Iod, (**b**) NPG/Iod, (**c**) Coum/NPG, and (**d**) Coum/Iod/NPG. The asterisk (*) denotes the excited state of the molecules.

Lalevée et al. studied in depth the mechanism of trimolecular systems [99], concluding that its advantage over bimolecular systems is its ability to convert terminating species (PHI**^·^** radicals) into initiating species (coI_(-H)_**^●^** and A**^●^**), which increases the yield of the initiating species (see Figure 12). Another valuable aspect of this system, highlighted by Lalevée, is the regeneration of the PI. The performance of the PI as a photocatalyst (PC) allows the use of low light intensity as well as low amounts of PI.

In a more recent work, Lalevée et al. established the mechanism for the trimolecular photoinitiating system Coum/Iod/NPG [88], as shown in Figure 12. Separately studying each of the three biomolecular combinations: Coum/Iod, Iod/NPG, and Coum/NPG (Figure 12a–c), they postulated a mechanism based on the analysis of these systems and the active species obtained from them (Figure 12d).

The first mechanistic pathway (Figure 12a) is exactly the same as in the bimolecular system, i.e., upon absorption, the excited coumarin (Coum*) transfers an electron to the Iod (the acceptor agent). This transfer is allowed from the singlet state as well as from the triplet state. Then, the iodonium salt (Ar_2_I^+^) decomposes into an aryl radical (Ar**^●^**) and aryl iodine. The formation of this radical (Ar**^●^**) was confirmed by detection of the radical adduct Ar**^●^**/NPG (*N*-phenylglycine) by ESR-ST (electron spin resonance spin trapping) experiments.

In the second mechanistic pathway (Figure 12b), the formation of a Charge Transfer Complex (CTC) [88,100] between NPG (donor component) and iodine (acceptor component) was proposed. This complex might also release the aryl radicals, since some photopolymerizations were carried out with high conversions using this bimolecular system.

The third mechanistic pathway (Figure 12c), also follows a bimolecular system. The interaction of the excited coumarin (Coum*) with NPG leads to the generation of a radical. This radical is obtained following the next sequences: (a) PNG transfers an electron to Coum*, (b) then a proton, and (c) in the last, step it undergoes decarboxylation. Since the decarboxylation step is irreversible, this radical might be considered as the initiating species in the trimolecular system. 

After this analysis and doing a recombination of the active species, the mechanism postulated by Lavelée et al. for the trimolecular system is described in the fourth mechanistic pathway (Figure 12d). The first initiating radical, generated from Coum/NPG system, reacts with the iodonium salt to generate a cation (NPG_(-H;-CO2)_^+^) and the aryl radical (Ar**^●^**). On the other hand, the protonated coumarin radical also reacts with the iodonium salt regenerating the PI (Coum) and yielding an aryl radical (Ar**^●^**) and a proton. Therefore, this trimolecular systems is able to catalyze free radical polymerizations as well as cationic polymerizations.

## 3. Polymers with Coumarin as Nonreactive Moiety in Electro-Optical Applications

Due to the tunable fluorescence and absorbance properties [101] coumarin molecules are incorporated in numerous application fields such as OLEDs [31,32,33,34], optical data storages [35,36], or laser dyes [37,38]. Trenor et al. and Wagner et al. published detailed reviews of coumarin moieties and their photophysical properties some years ago [29,102]. Therefore, the following chapters will discuss the recent trends in coumarin containing polymers for different electro-optical applications.

### 3.1. Fluorescence Studies

Researchers have been interested in the photophysical properties of coumarin moieties since the early 1940s, relying on their versatile absorbance and fluorescence [29,101]. In the late 1950s, the group of Wheelock demonstrated a shift of the fluorescence band by substitutions on the coumarin structure [29,30]. Nearly 60 years ago, they investigated that additional electron-repelling groups in positions 4, 6, or 7 or electron-attracting groups in position 3 result in a shift of the fluorescence band to longer wavelengths and that a substitution of the carbonyl with a thione red shifts the absorbance and quenches the fluorescence [30]. Apart from the substitution of coumarin, the used solvent [101,103] and the pH value [104] of the solution also affect the absorbance and fluorescence spectra. By increasing the pH of the solution, the fluorescence intensity increases [104], and by increasing the polarity of the solvent, the absorbance of coumarin derivatives is red shifted, whereas the emission of the coumarin moieties is broadened and shifted to higher wavelengths due to reinforced hydrogen bonding [101]. In 1970, Song and Grodon published an extended spectroscopic study of coumarin itself and discovered that the fluorescence emission relies on a ^1^(π,π*) excited state, which is red shifted in nonpolar solvents [103].

**Figure 13:**
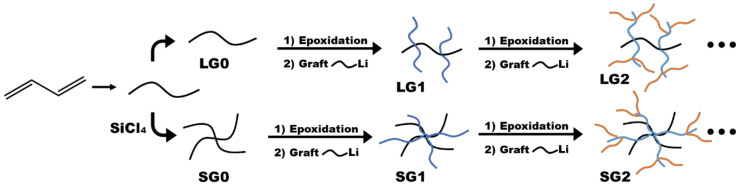
Synthetic routes of dendrigraft polybutadienes with linear-comb or star-comb architecture from generation 0 to 2. Figure adapted from [107].

These publications have become the starting point to incorporate coumarin moieties in different polymer systems in order to use and study their versatile fluorescence properties. Recently, Zhang et al. synthesized multicoumarin-functionalized dendrigraft polybutadienes from generation 0 to 3 in linear or star shape to determine the dendritic effects on the spectroscopic properties (Figure 13). They observed a hypsochromic shift (blue shift to lower wavelengths) of absorption and emission maxima in combination with signal amplification for the third-generation dendrimers (both shapes) due to the encapsulation effect of the compact persistent structure. In lower generation dendrimers, the dendritic effect on the fluorescence was enhanced (positive dendric effect), whereas at higher generation, a negative dendritic effect could be measured as a result of synergistic effects of inter-/intramolecular fluorescence quenching. Moreover, linear dendrimers with an easy topology enabled better fluorescence properties compared to their star-shaped analogues [105].

Tocco et al. linked coumarin moieties to poly(ethylene glycol) via an ether spacer to make it more resistant to chemical and physical damage and to increase the solubility of coumarin. The anchored coumarin showed similar luminescence compared to the molecular one despite the large spacer [106]. In another approach, Teixeira et al. reported on the copolymerization of 3-vinylcoumarins and 7-hydroxy-3-vinylcoumarin with styrene and methyl acrylates and the resulting spectroscopic changes [34]. They discovered a bathochromic shift of the UV absorption maximum of 3-vinylcoumarin compared to 7-hydroxycoumarin due to the extension of the conjugated system. Due to polymerization, the vinyl-group is saturated and emission and absorption maxima were shifted to lower wavelengths. Moreover, a loss in total quantum yield was obtained for all co-oligomers in comparison to the vinyl-functional coumarin monomers.

The fluorescence of coumarin and its derivatives are used in many different application fields such as in bioimaging [107,108,109,110,111,112], the production of optical fibers by dye doping of PMMA fibers [37], ion detection [113], or damage monitoring in epoxy adhesives under UV light [114]. Another interesting application field of coumarin moieties was shown by Zhang et al., who synthesized polymeric fluorescent brightener based on coumarin for the paper industry. The functional polymer was soluble in water, increased the surface strength and smoothness of paper as surface sizing agent, and inhibited UV-aging as fluorescent brightener and light stabilizer. The synthesized polymer showed a smaller Stokes shift than the monomeric fluorescence brightener, suggesting a better photostability and smaller energy loss due to steric hindrance [115]. The same group also prepared a multifunctional fluorescent polymer by copolymerization of a fluorescent coumarin-containing monomer and an ultraviolet absorber monomer. The synthesized multifunctional polymer exhibited comparably good results regarding surface sizing, fluorescent brightening, light stabilizing, and water solubility [116].

**Figure 14:**
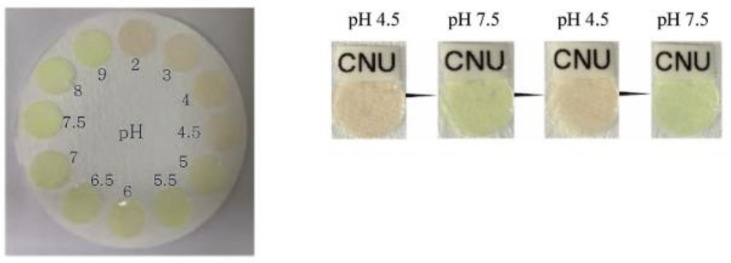
Visual color transition of the MF-C6-Nf membrane at different pHs (pH 2, 3, 4, 4.5, 5, 5.5, 6, 6.5, 7, 7.5, 8, and 9) under sunlight and images of reversible color of the MF-C6-Nf membrane when it was exposed to pH 4.5 and 7.5 under sunlight. Reproduced with permission from [119]. Copyright 2020©, Elsevier.

Furthermore, coumarin moieties are used in the preparation of pH-sensors [38,117,118]. For example, coumarin dyes were physically incorporated into melamine-formaldehyde resin particles (MF-C6) and subsequently attached to Nafion hydrogels (MF-C6-Nf) to produce pH-sensing membranes (Figure 14). They enabled the measurement of pH values from 4.5 to 7.5 by a shift in the fluorescence emission spectra and a color transition under visible light from pink to yellow. The membranes were highly sensitive, reversible, as well as stable and could be applied in pH value measurements of real urine samples and fermentation media [117].

Moreover, membranes with covalently attached coumarin derivatives were prepared by synthesizing polymerizable coumarin indicators. The covalently immobilized molecules monitored pH changes in the alkaline region by decreasing fluorescence intensity with increasing pH value and featured good photostability and photobleaching performance [118]. Additionally, Albertazzi and coworkers synthesized dendrimer-based fluorescent biosensors with different dyes for targeted delivery in living cells and the sensing of pH values from 6 to 10 [38]. Depending on their size and surface charge, the dendrimers displayed specific subcellular localization. By conjugation of pH-sensitive and insensitive molecules, ratiometric pH sensors were prepared and calibrated in vitro and in living cells, which enabled selective pH measurements in different parts of living cells.

### 3.2. Electroluminescence Studies

**Figure 15:**
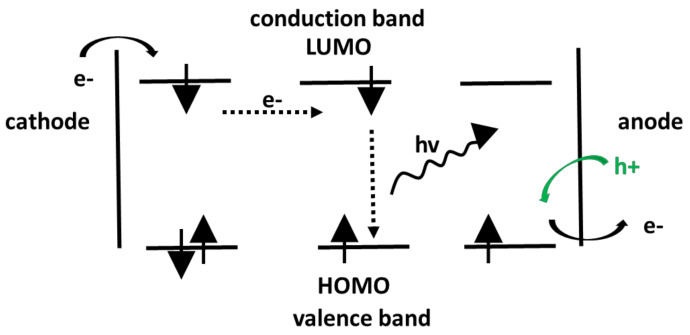
Schematic presentation of electroluminescence. Figure adapted from [121].

In electroluminescence, electrical energy is directly converted into light, whereas photoluminescence is the direct conversion of UV light into visible light (Figure 15). By placing a conjugated polymer between two electrodes, electrons are injected into the lowest unoccupied orbital (LUMO) from the cathode while the anode extracts electrons from the highest orbital (HOMO) via an injection of holes in the HOMO level of the polymer. If an electric field is applied, electrons and holes move within the polymer to the other electrode and are able to recombine and form an exciton, which emits light through exceeding the ground state [119]. Conjugated polymers with electroluminescent properties have gained high research interest due to their high efficiency, lifetime, and luminance [120], which can be easily tuned throughout the visible light spectrum [29].

Due to luminescence and mechanical properties, polyphenylene vinylenes (PPVs) are one of the most well-studied conjugated polymer classes. Huang et al. synthesized different coumarin-terminated PPV-systems following the Gilch methodology to prepare light-emitting diodes with yellow emission. The modification of the conjugated polymer resulted in a blue-shifted emission color and improved luminescent efficiencies compared to conventional PPV systems [120]. Another interesting conductive polymer class are carbazole-based macromolecules. Due to easy modification at their (3,6-), (2,7-), and *N*-position, polymers with broken conjugation at their 3,6- position showing multicolored electrochromic properties can be synthesized. The band gap can be further decreased by the functionalization of carbazole units with various electron-donating groups at the 2,7- or 3,6- position. Yigit and his group studied this phenomenon by synthesizing four new 3,6-linked thiophene-carbazole based polymers bearing strong chromophores such as coumarin and azobenzene via Stille cross-coupling reaction [121]. The results suggested a remarkable influence of the type of chromophore on electronic and optical properties. Coumarin-containing polymers showed better electrochemical and optical properties in terms of band gaps, optical contrast, and switching times compared to the azobenzene containing analogues. However, compared to other conventional conductive polymers with coumarin and azobenzene groups, the synthesized ones had lower band gaps and better multichromic properties with a broader color spectrum.

**Figure 16:**
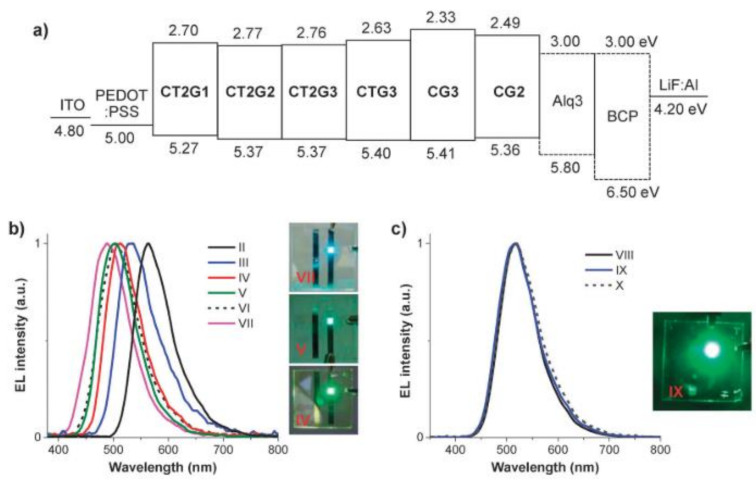
Schematic (**a**) energy diagram of the fabricated organic light emitting diodes (OLEDs). (**b**,**c**) Emission light spectra of the OLEDs (devices II–X) fabricated with carbazole dendronized coumarin derivatives as an emissive layer and a hole transporting layer and their device emission colors. Reproduced with permission from [32]. Copyright 2014©, Royal Society of Chemistry, RSC.

Recently, the group of Promarak studied the electroluminescence characteristics of coumarin containing carbazole dendrons consisting of thiophenyl [33] or oligothiophenyl [32] coumarins as cores and carbazole dendrons up to the third generation as substituents, to prepare nondoped solution-processed light emitter and hole transport layers for OLEDs (Figure 16). The crystallization was reduced, the high emission of coumarin cores in the solid state was retained and the amorphous stability of the material was improved using carbazole dendrons as substituents. Used as emissive layer, the solution processed OLEDs showed light blue to yellow colors, whereas when used as hole-transporting layers, bright green emission could be observed. The color was changed by varying the number of thiophene units or the number of generations of the dendrons. In particular, the third-generation macromolecule showed a good performance as light emitter and hole-transporting layer [32,33]. Subsequently, the same group synthesized novel dendrimers based on oligothiophenyl *N*-coumarins as fluorescent core and carbazole dendrons as substituent. Using dendrimers as substituent, the aggregation-caused emission quenching of the *N*-coumarin core could be prevented, and the hole-transport ability, thermal stability, and solubility were increased [31].

### 3.3. Light and Energy Harvesting

To harvest solar energy with the help of polymeric systems is a topic of high interest—in particular, the study of efficient energy transfer processes in polymer chains consisting of donor chromophores that absorb the light and subsequently capture the energy in acceptor moieties. Such polymers mimic the light-harvesting process of photosynthetic systems in green plants and can increase the light absorption cross-section [122]. In the early 1990s, Lang and Drickamer started to incorporate coumarin derivatives into poly-(acrylic acid) to study the harvesting and transfer of solar radiation energy between 7-dimethylaminocyclopenta[c]coumarin and rhodamine B [123].

By conventional free radical polymerization, it is difficult to obtain well-defined photoactive polymers, thus extensive research about alternative routes has been conducted. Chen et al. suggested reversible addition-fragmentation chain transfer (RAFT) polymerization to introduce chromophores into polymers in a suitable way, which can be applied to all classical radical polymerization systems. They introduced coumarin chromophores as energy acceptor molecules into a RAFT agent to polymerize the energy donor molecule, acenaphthylene, in a controlled way. Using a ratio of 1:1 between RAFT agent and monomer, each monomer radical was trapped by the RAFT agent and formed an intermediate radical despite propagating. Thus, each polymer chain was end capped with a coumarin moiety. In aqueous solutions with pH 11, the polymers showed 100% energy transfer efficiency due to reduced interchromophore separations and the maximum of coumarin emission was red shifted to 455 nm [124].

Recently, researchers have focused on the synthesis of dendrons for energy harvesting applications. The group of Knoester studied the energy transfer process in a first-generation coumarin-tetraphenylporphyrin donor-acceptor dendrimer. Fast energy transfer kinetics (500 fs) and high efficiency (97%) could be obtained due to the presence of multiple donor molecules [125]. Another group examined coumarin-perylene bisimide first-generation dendrimers and observed a fast (10 ps) and efficient (99.5%) energy transfer at low donor excitation density [126]. At higher excitation densities (more than 1 absorbed photon per 10 dendrimer molecules), the transfer rate increased, based on excitation of multiple donors per dendrimer. By pre-excitation of the acceptor, the donor–acceptor energy transfer rate could be controlled. Aydinli et al. prepared poly(aryl ether) dendrimers up to the second generation with 4-methyl-7-hydroxycoumarin as a donor on the surface and 3-cyano-7-hydroxycoumarin as an acceptor at the focal point to investigate the energy transfer efficiency in higher generation dendrons. By increasing the generation of the dendrimer, both the number of peripheral chromophores and the absorption and emission increased. However, the energy transfer from the periphery to the core occurred nearly quantitative [127]. More recently, Mao and Song have synthesized three different porphyrin-cored dendrimers with coumarin-moieties in the periphery and investigated two factors that influence the energy transfer efficiency. First, the better the spectral overlap between the absorption spectrum of porphyrin and emission spectrum of coumarin, the higher the energy transfer efficiency occurs. Second, long alkyl side-chains prevent coumarin derivatives from self-quenching and, therefore, dendrimers with *N*-octyl groups showed higher efficiencies than those with *N*-ethyl groups [128].

### 3.4. Liquid Crystalline Polymers

Polymeric liquid crystals combine polymer-specific properties with the anisotropic behavior of liquid crystalline molecules. By introducing liquid crystals (LC) into polymers, the mechanical strength can be increased and the LC phase behavior changes [29]. Coumarin moieties were introduced into liquid crystals either to use the photodimerization reaction for photoalignment of liquid crystals or to exploit their fluorescence properties. Cavero et al. synthesized poly(propylene imine) (PPI) dendrimers with meomorphic cyanobiphenyl units and cinnamate- or coumarin-based photoactive molecules in the periphery to reduce the recrystallization affinity of the final material [129].

One special class of polymeric liquid crystals are photoactive liquid crystalline dendrimers. Romero and coworkers started to study liquid crystal dendrimers relying on hydrogen bonding between an s-triazine central core and three peripheral dendrons consisting of bis(hydroxymethyl)proprionic acid in 2014 [130]. Amongst others, they synthesized asymmetric dendrons with achiral promesogenic units combined with coumarin or pyrene as an active moiety. For symmetric dendrons and their complexes, smectic properties could be observed, whereas the asymmetric ones possessed nematic characteristics. The absorption behaviors of the structures were studied in thin films and in solution. In thin films, a broadening and blue shift of the highest absorption band was monitored due to the packing effect of coumarin in condensed phase and dye–dye interactions. The emission spectra also displayed a broadening and a red shift at 530 nm (compared to 455–458 in solution) in thin films based on aggregation of the coumarin units. Compared to dendrons, dendrimers revealed a higher photoconductivity, which can be related to the presence of triazine. On the one hand, triazine moieties increased the electron mobility and on the other hand, the efficiency of photogeneration could be increased due to interactions between triazine and coumarin. In a follow-up study, they synthesized liquid crystalline dendrimers based on hydrogen bonding between a porphyrin—Zn complex in the core and four peripheral carboxylic acid dendrons. The asymmetric dendrons and dendrimers derived from a promesogenic unit and coumarin as an active molecule did not have any liquid crystalline-like behavior since in the porphyrin core, no mesomorphic phase could be observed [131]. In a subsequent study, they investigated dendrimers with a porphyrin-Zn/Cu core, which was covalently linked to four coumarin containing dendrons [132]. Those dendrimers formed nematic discotic mesophases (N_D_) with high tendency forward homeotropic alignment. Due to their spontaneous alignment between electrodes, easy synthesis, and self-healing capability, they were suitable candidates for OLEDs, organic field-effect transistors (OFETs), and organic photovoltaic devices (OPVs). Typically, supramolecular organization in columns is used to achieve high charge mobility. However, Concellón et al. observed as high values in nematic discotic mesophases as in ordered columnar mesophases. Owing to a lower degree of order, large uniform domains can be formed easily in N_D_, while no long-range positional order is possible [132].

Recently, the same group has synthesized another new type of liquid crystalline porphyrin-core dendrimers with coumarin moieties in the periphery via the copper-catalyzed azide-alkyne “click” cycloaddition (CuAAC). Owing to the coumarin units, discotic nematic mesophases with hole mobility values comparable to the highest values reported for discotic liquid crystals could be obtained. By exciting the coumarin moieties, energy transfer to the luminescent porphyrin core was monitored (antenna effect) which was faster with increasing generation of the dendrimer. The second-generation dendrimer featured not only better optical (antenna effect) properties but also more efficient electronic properties (hole mobility) than the first-generation analogue [133].

## 4. Sustainable Polymers with Coumarin as Nonreactive Moiety

Plastic pollution is one of the biggest environmental problems, since every year, nearly 8 million tons of plastics end up in the oceans. Although the production of plastics based on fossil fuels is steadily increasing [134] research efforts are placed on the development of bio-based and biodegradable polymers to overcome the dependence on fossil fuels and to lower plastic pollution [135]. As coumarin can be isolated from natural resources [3], it is incorporated into polymers to increase the sustainability.

**Figure 17:**
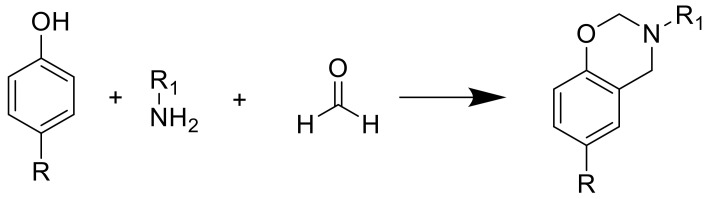
Synthesis of benzoxazines.

Recently, polybenzoxazines have been commercialized thermosetting resins with varying properties, such as low shrinkage during polymerization, low water absorption, and molecular structure variability, as benzoxazines are synthesized from a phenol part, a primary amine and formaldehyde (Figure 17) [136].

**Figure 18:**
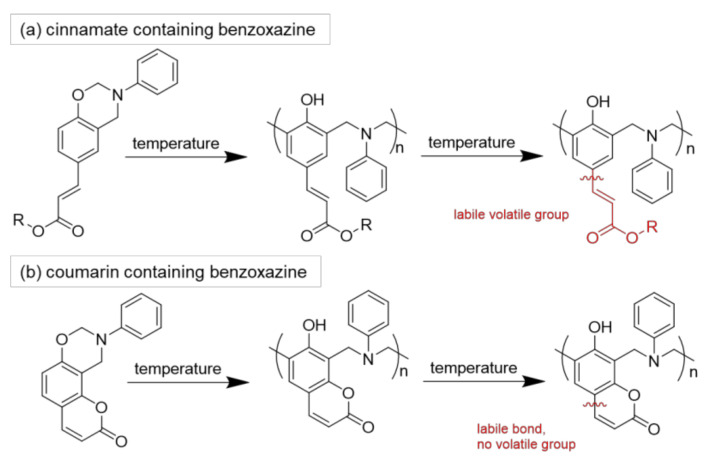
Possible degradation mechanism in (**a**) cinnamate and (**b**) coumarin containing benzoxazines during polymerization at elevated temperatures [138].

Up to now, commercially available benzoxazines are petroleum-based but there is a major research interest in using renewable resources as amine and/or phenolic part [137]. For example, cardinol, cinnamic/cinnamates acid, eugenol, vanillin, or groups of cellulose were used in order to produce benzoxazine resins. However, the main issue of bio-based polymers is their reduced thermal stability due to the formation of volatile groups with increasing temperature during polymerization (Figure 18). Froimowicz et al. synthesized a thermally stable benzoxazine monomer based on umbelliferone (7-hydroxycoumarin) in 2015 [138]. They reported an increasing thermal stability (>300 °C) of partly bio-based benzoxazines using a coumarin moiety as phenolic compound due to a ring-opening reaction instead of the formation of volatile groups at higher temperatures. The resin homopolymerized without a catalyst to lower the polymerization temperature and showed comparable performance to petroleum-based analogs with respect to design, synthesis, and applicability.

In a subsequent work, Froimowicz and his group studied the influence of substituents on the coumarin-based benzoxazines regarding their reactivity and thermal properties. They used phenol (PH-a), umbelliferone (U-a) and 4-methylumbelliferone (MU-a), and aniline to synthesize three different resins and compared their polymerization rate/temperature and thermal stability. Generally, umbellliferone moieties accelerate the polymerization and lower the polymerization temperature due to the conjugated C=C double bond in the ring. However, electron-donating substituents (on the double bond) lower the electrophilic behavior of the carbon double bond and increase the polymerization rate/temperature (see Table 1) [139].

**Figure 19:**
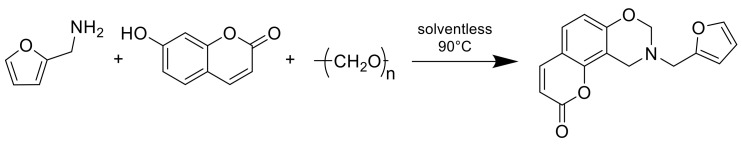
Synthesis of the first fully bio-based benzoxazines.

In 2016, the first fully bio-based coumarin containing polybenzoxazine was synthesized using umbelliferone, furfurylamine, and formaldehyde as renewable resources. Froimowicz and coworkers demonstrated three different synthetic approaches—the solventless method being the most ecofriendly method (Figure 19). Due to the incorporation of a furfurylamine moiety a “cooperative activation effect” was induced by the electrophilic C=C double bond of the coumarin ring and the aliphatic amine. The conjugated double bond activates the polymerization, while the furan ring subjects electrophilic substitution, which result in lower polymerization temperatures, a higher cross-linking density, and improved thermal stability [140].

Liu et al. synthesized new fully bio-based benzoxazines from rosin. Dehydrobietylamine can be extracted from rosin and, in combination with 4-methylumbelliferone or guaiacol and paraformaldehyde, thermally stable and anticorrosive coatings can be prepared [23].

Another possibility to make commercial thermosets greener and tougher is a double-network approach. The blending and copolymerization of plant oils and their derivatives with epoxy resins is a common way to toughen commercially available epoxy systems. While the impact strength can be improved, the tensile strength decreases due to insufficient interfacial interactions between epoxy resins and plants oils [141]. Cai and coworkers used coumarin as a building block for one network and diglycidyl ether of bisphenol A (DGEBA) for the second one. The good interfacial adhesion between the two networks resulted in only one glass transition temperature, an improved toughness and no loss in tensile strength and elongation at break [142].

**Figure 20:**
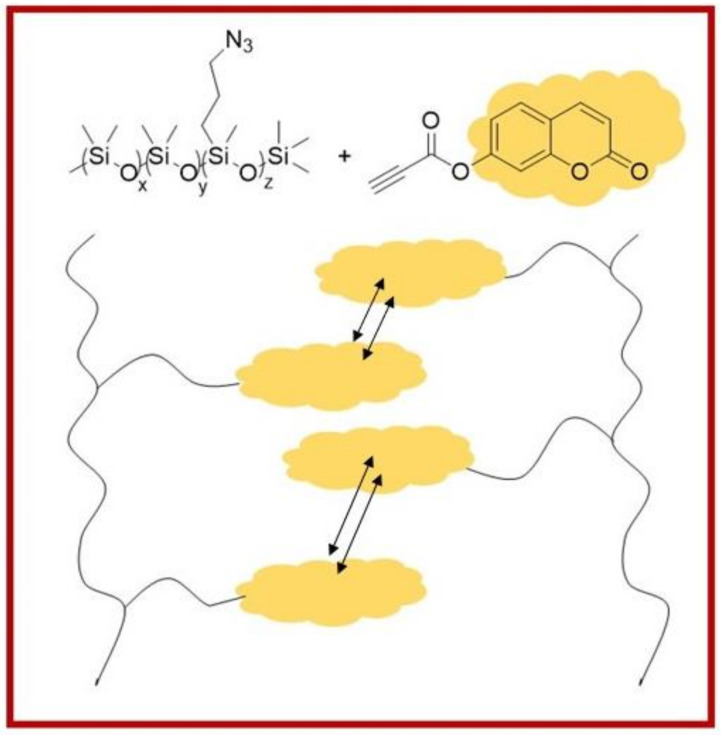
Coumarin as functional groups in thermoplastic materials.

Coumarin is not only used in thermosets but also in thermoplastics. Fawcett and coworkers incorporated different concentrations of coumarin moieties in the backbone of silicone polymers that resulted in a physical silicone polymeric network with thermoplastic elastomeric characteristics, although silicone elastomers are normally thermosetting materials (Figure 20). Without covalent cross-linking, only by head-to-tail π-stacking of the coumarin molecules to the silicone chains, the mechanical properties can be tailored [143].

## 5. Biological and Medical Applications of Polymers with Coumarin as Nonreactive Moiety

### 5.1. Antimicrobial Coatings

Increasing bacterial infections and antimicrobials resistances are growing problems in the public health system. Bacterial contamination of surgical tools, implants, catheters, and surfaces in hospitals causes infections and underline the need of antimicrobial polymeric coatings [10]. Due to the antimicrobial [17,18,19,144,145,146], antifungal [3], and antibacterial [3,10,11,12,13,14,15,16,147] activity of coumarin moieties, it is an interesting molecule for biological active polymeric coatings.

**Figure 21:**
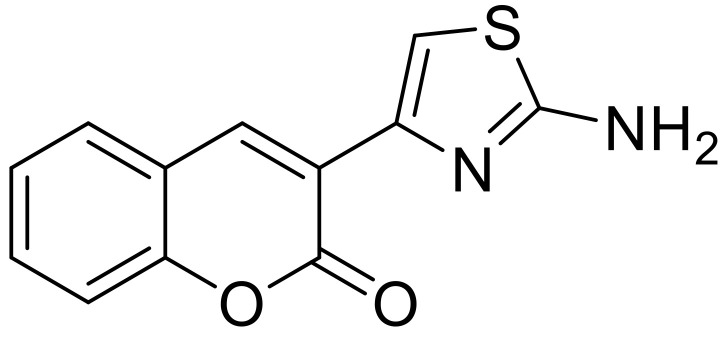
Structure of 2-(2-amino-1,3-thiazol-4-yl)-3H-benzo[f]chromen-3-one.

Recently, researchers have started to incorporate coumarin derivatives into polyurethane coatings to enhance their antimicrobial activity [144,145]. El-Wahab et al. synthesized a coumarin thiazole moiety 2-(2-amino-1,3-thiazol-4-yl)-3H-benzo[f]chromen-3-one (Figure 21) and mixed it with a polyurethane coating based on sunflower oil, glycerol, pentaerythritol, toluene diisocyanate, and turpentine. To determine the antimicrobial activity of the polyurethane, different bacteria, and fungi were used and the coating showed better performance against Gram-positive bacteria. The increased antimicrobial effect was attributed to the coumarin and the thiazole ring [144].

**Figure 22:**
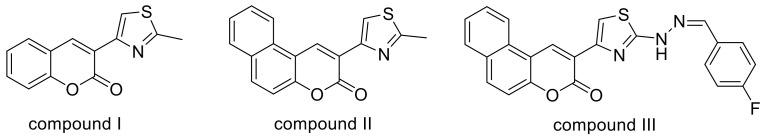
Structures of the synthesized coumarin thiazole derivatives.

In a subsequent study, the same group synthesized three other coumarin thiazole derivatives (Figure 22) for polyurethane coatings to observe their antibacterial, flame retardant, and anticorrosion properties. The amount of coumarin in the coatings varied (0.5–1.5 wt.%) and the coatings were applied on steel and wood panels for the testing against Gram-negative and Gram-positive bacteria and fungi. It could be shown that the antimicrobial activity increased with rising amount of the coumarin moieties.

Apart from the antimicrobial activity, the flame retardancy of polyurethane coating improved as well. The limiting oxygen index (LOI) of polyurethane was 20 and increased to 43 by incorporating 1.5 wt.% of compound 2. The flame retardant efficiency and the antimicrobial activity of the synthesized derivatives decreased in the following order: compound II > III > I [145]. Jaiswal et al. incorporated silver nitrate and carboxylated coumarin—silver complexes into sol–gel materials to prepare coatings on glass slides and in microtiter wells. The antimicrobial performance of the coatings was studied with *Staphylococcus epidermidis* CSF 41498 and the cytotoxicity for human keratinocyte cells was investigated. Ag^+^ ions were gradually released from the coating over a prolonged period that increased the antimicrobial effect. The carboxylated coumarin facilitated the release of the Ag^+^ ions and created a less cytotoxic surface compared to silver coatings without coumarin [147].

**Figure 23:**
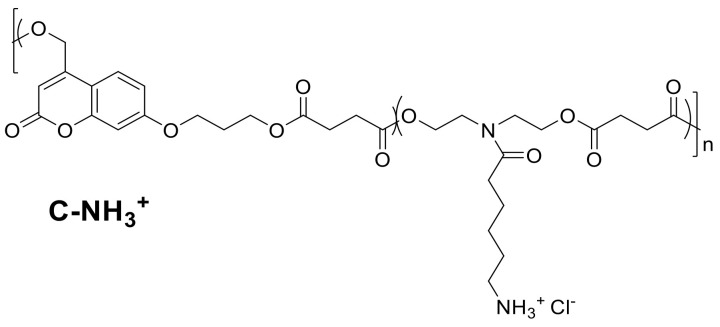
Structure of the cationic coumarin polyester.

However, coumarin derivatives cannot only be used as an additive in coatings, but also be part of the polymeric backbone. In 2017, Chamsaz et al. prepared cationic coumarin polyester coatings (Figure 23), which showed a good antimicrobial activity against *Pseudomonas aeruginosa* biofilm formation.

**Figure 24:**
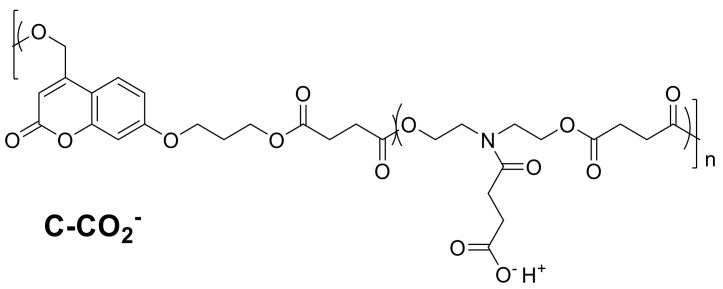
Structure of the synthesized anionic coumarin polyester.

Polyesters with cationic amine groups were coated onto glass substrates and their efficiency against Gram-negative bacteria colonialization on the surface was evaluated. The cationic coumarin polyester killed the bacteria on the surface and avoided biofilm formation but was not hemolytic active or discernible toxic towards mammalian cells. For comparison, the same group also synthesized an anionic polyester (Figure 24), which was not antimicrobial active, therefore, the activity of the cationic one can be attributed to the cationic charge and not to the coumarin moiety in the backbone [10].

### 5.2. Biologically Active Polymers

Polymers with reactive functional groups are not only used for their macromolecular properties but also for the properties of their functional groups. Additional functional groups enable a subsequent modification of the polymers for more specific applications [148]. As the introduction of coumarin into the backbone or side chain of a polymer results in a broad variety of applications, researchers have already started to synthesize coumarin containing biologically active polymers in 1970 [149].

**Figure 25:**
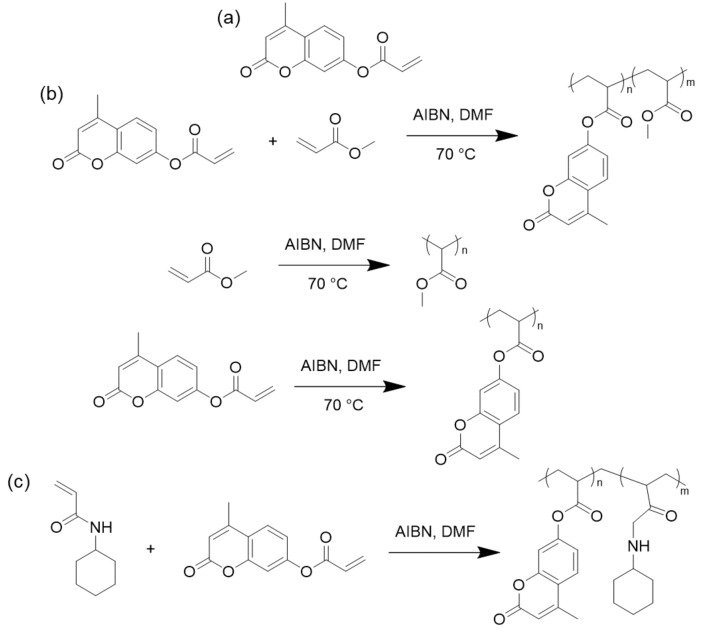
(**a**) Structure of 7-acryloyloxy-4-methyl-coumarin (AOMC), (**b**) synthesized (co)polymers with methyl acrylate, and (**c**) synthesized copolymer with NCA.

In 2008, Patel and coworkers synthesized 7-acryloyloxy-4-methyl-coumarin (AOMC) (Figure 25a) and its homo- and copolymers with methyl acrylate (Figure 25b). The polymers were moderately thermally stable and by variation of the composition, the thermal stability, rate of decomposition, and activation energy changed. The antimicrobial activity of the copolymers was evaluated against different bacteria, fungi, and yeasts and increased with increasing coumarin content [19]. Chitra and coworkers prepared AOMC and copolymerized it with *N*-cyclohexylacrylamide (NCA) (Figure 25c) in various ratios. The antifungal activity was investigated with *Aspergillus flavus*, *Candida albicans,* and *Candida tropicalis* fungi and the antibacterial efficiency was obtained using *Escherichia coli*, *Salmonella typhi,* and *Bacillus cereus*. The results showed that biological activity increases with an increase in NCA in the copolymers [13].

**Figure 26:**
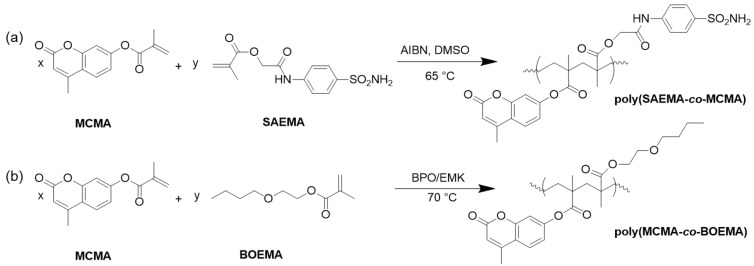
(**a**) Copolymerization of 4-methyl-2-oxo-2H-chromen-7-yl-2-methylpropenoate (MCMA) with 2-oxo-2-[(4-sulfamoylphenyl)amino]ethyl-2-methylpropenoate (SAEMA) in DMSO at 65 °C and (**b**) copolymerization of MCMA with butoxyethyl methacrylate (BOEMA) in ethyl methyl ketone at 70 °C.

Erol and coworkers synthesized a coumarin-containing methacrylate copolymer via free radical copolymerization of 2-oxo-2-[(4-sulfamoylphenyl)amino]ethyl-2-methylpropenoate (SAEMA) with 4-methyl-2-oxo-2H-chromen-7-yl-2-methylpropenoate (MCMA) (Figure 26a). DSC measurements indicated an increasing glass transition temperature with rising MCMA content.

The antimicrobial activity of the synthesized homo- and copolymers was investigated using *Pseudomonas aeruginosa*, *Escherichia coli*, *Proteus vulgaris*, *Salmonella enteridis*, *Klebsiella pneumonia*, *Staphylococcus aureus*, and *Candida albicans*. Due to the peculiar chemical structure of the copolymers with mutual lipophilic and hydrophilic groups of the sulfonamides, the polymers were moderately active and comparable to standard drugs like penicillin G and teicoplanin. Thus, the activity of the copolymers would allow the design of surfactants and antimicrobial systems [12]. Venkatesan et al. copolymerized MCMA with butoxyethyl methacrylate (BOEMA) in different ratios via free radical polymerization in ethyl methyl ketone at 70 °C with benzyl peroxide (BPO) as initiator (Figure 26b). Thermal stability, glass transition temperature, and antibacterial activity increased with rising MCMA content. Due to the electron-rich and electron-poor centers of coumarin’s benzene ring, microorganism can attack these centers (depending on their nature) and their growth is inhibited. The antibacterial activity was studied against selected bacteria (*P. aeruginosa*, *S. aureus*, *K. pneumoniae*, *B. subtilis*, *B. cereus*, and *K. planticola*) and compared with ampicillin [16].

**Figure 27:**
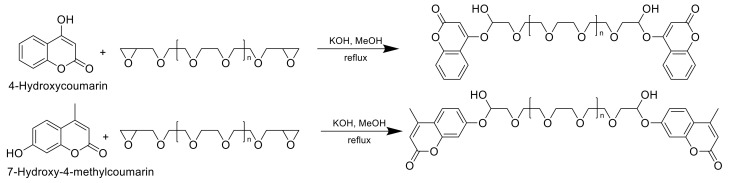
Functionalization of oligoethylene glycol diglycidyl ethers with 4-hydroxycoumarin and 7-hydroxy-4-methylcoumarin.

Another group incorporated 7-hydroxy-4-methyl-2H-chromen-2-one and 4-hydroxy-2H-chromen-2-one into oligoethylene glycol diglycidyl ethers with different chain lengths (Figure 27). The activity was tested in vitro against Gram-positive bacteria *Staphylococcus aureus* and *Bacillus cereus* and Gram-negative salmonella and clearly indicated a dependency on the chemical structure of the compounds. The polymers functionalized with 7-hydroxy-4-methylcoumarin showed a higher antimicrobial activity than the others, but, nevertheless, all of the tested compounds had moderate activity against the bacteria and could be effective antibacterial agents [14].

**Figure 28:**
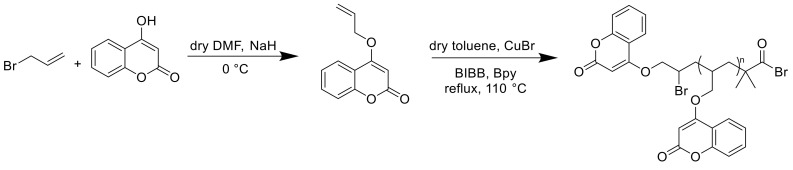
Synthesis of 4-allyloxycoumarin and its polymerization via atom transfer radical polymerization (ATRP).

Another interesting work was published by Srivastava et al., who reported the synthesis of a polymer containing 4-allyloxycoumarin (ACO) via atom transfer radical polymerization (ATRP) in toluene at 110 °C with 2-bromoisobutyryl bromide (BIBB), Cu(I)Br, and 2,20-bipyridyl (bpy) as initiator and catalyst (Figure 28). The polymer was weakly active against *Enterococcus faecalis* but by synthesizing a silver nanocomposite, the antibacterial activity could be improved [15].

Furthermore, coumarin derivatives show anti-inflammatory [22,150] and antioxidant [17,20,21,22,151,152] properties. In 2007, Pandey et al. synthesized PEGylated 4-methylcoumarins to improve the antioxidant properties of 4-methylcoumarin and to increase the hydrophilicity for a broader application field [152]. Subsequently, the same group synthesized PEGylated 4-methyl as well as 4,8-dimethylcoumarins and evaluated their anti-inflammatory characteristics for ICAM-1 (intercellular cell adhesion molecule-1) inhibition on human endothelial cells. The synthesis was performed in two different ways: first, a solvent-free enzyme (*Candida antarctica* lipase) catalyzed a copolymerization of the diesters of 4-methyl- and 4,8-dimethylcoumarin with poly(ethylene glyocol), and second, a bromination of 4-methyl and 4,8-dimethylcoumarin was carried out with the subsequent attachment to already synthesized PEGylated polymers. The synthesized products showed higher anti-inflammatory efficiency than the non-PEGylated ones, which was explained by their improved solubility in aqueous and organic media [150].

**Figure 29:**
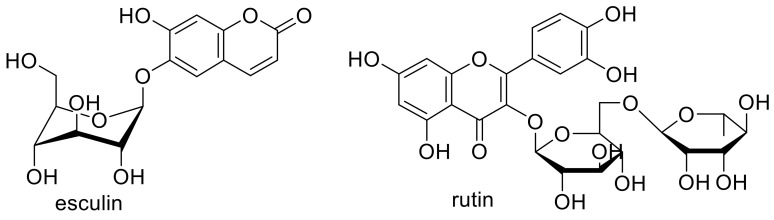
Structure of esculin and rutin.

Chebil et al. performed an enzymatic polymerization of rutin and esculin (Figure 29) and evaluated the antioxidant performance of polyrutin and polyesculin.

By analyzing the rutin polymers via FTIR-spectroscopy, new C-C and C-O bonds were observed, while in polyesculin, only new C-C bonds were formed. This resulted in a decreasing antioxidant activity with increasing polymerization degree of rutin and a highly increasing activity of polyesculin compared to monomeric esculin. The antioxidant activity showed a dependence on the position of linkage through the polymerization reaction but both polymers featured a high XO inhibition activity, iron chelating, and cupric reducing antioxidant capacities [20].

Recently, Li and coworkers improved the antioxidant activity of chitosan by incorporating coumarin moieties in a three-step reaction. The inhibitory performance of the synthesized chitosan derivatives was remarkably improved over conventional chitosan and the antioxidant activity was also increased. The modified chitosan derivatives did not show any cytotoxicity and promoted the cell growth due to their high antioxidant activity, which enables the usage of the synthesized chitosan polymer in different biomedical areas [21].

### 5.3. Gene and Drug Delivery

Polymers for biomedical and pharmaceutical applications have raised the interest of numerous research groups in the past decades. They can be used in drug delivery systems, as scaffolds for tissue-engineering and repairing and new biomaterials [153]. Particularly, drug delivery systems have gained a high impact because of their ability to guide drugs precisely, lower side effects and/or keep drugs at a lower concentration over a longer period in the body by controlled release reactions [154]. To realize these advanced applications, well-defined polymers with tailorable properties and different architectures have to be synthesized. One possibility to synthesize specific building blocks for medical and biological application are click reactions [153].

In 2001, Sharpless et al. introduced the term “click reaction” to merge a set of reactions that fulfill several criteria, such as performance at ambient or mild conditions, modular, high yielding, missing, or harmless side products, and preferred by many different functional groups to enable the use of readily available educts [155]. Wu and coworkers used the copper(I)-catalyzed azide-alkyne cycloaddition click reaction to synthesize unsymmetrical dendrimers from mannose binding units and coumarin fluorescent units. Using this click reaction, they were able to introduce three different functional groups into the drug delivery system to provide a targeting moiety, a medicinally active agent (drug) and a diagnostic label such as coumarin at a specific position within the dendrimer. The dendrons with unique acetylene and azide groups at the focal point were linked via a stable [1,2,3]-triazole ring and the sequential modification of the chain ends resulted in the introduction of mannose and coumarin moieties at the periphery of individual blocks. The dendrimers were highly efficient recognition and detection agents for the inhibition of hemagglutination [156].

Apart from dendrimers, other nanoaggregates can also be used for drug delivery. Behl et al. synthesized three different coumarin—poly(ethylene glycol) (PEG) conjugates (PC1, PC2, and PC3) via the copper(I)-catalyzed azide-alkyne cycloaddition click reaction to introduce triazole moieties and to enhance the photophysical properties of coumarin. Triazole and coumarin rings are able to undergo π–π stacking interactions and, in combination with the hydrophilic-hydrophobic interactions, self-assembled nanoaggregates in the size of 100–120 nm with a negative free micellization energy (27 kJ/mol) were formed. The results of the photophysical characterization exhibit an increase in the quantum yield of PC2 and a decrease in the case of PC1 and PC3, due to the presence of a lower nonemissive S1-state that is noticeable by the large radiative lifetimes (35.7 and 42 ns) in the case of PC1 and PC3 compared to PC2 (3.83 ns). The π–π stacking interactions as a driving force for the formation of self-assembly were confirmed by the changes in fluorescence excitation spectra and 1H-NMR spectra. Since the polymers were biocompatible with human pancreatic cancer cells, the aggregates could be used as drug delivery systems to incorporate hydrophobic drugs [109].

**Figure 30:**
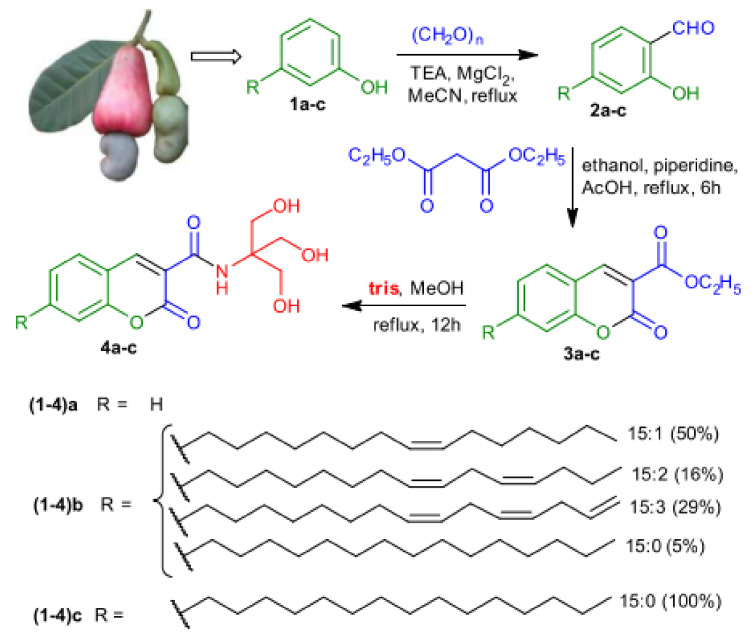
Synthesis of coumarin coupled tris amphiphile 4a-c. Adapted with permission from [112,157]. Copyright 2015©, Royal Society of Chemistry, RSC.

In general, self-assembly is a widely used method to generate drug delivery systems such as nanoaggregates or hydrogels. Lalitha and coworkers produced hydrogels via self-assembly of coumarin-tris derivates from renewable resources (Figure 30) [157]. They synthesized different coumarin-tris compounds with a varying hydrophobic part and a coumarin derivative as hydrophilic head. The amphiphilic compounds formed hydrogels in specific water to DMSO ratios after heating and sonication for a few seconds.

Compounds 4a and 4b showed stifling of the gelation ability due to a lack of hydrophobicity (in the case of 4a) and the kink in hydrophobicity (in the case of 4b), which is an indication for the importance of optimized hydrophilic and hydrophobic interactions to observe self-assemblies in macromolecules. ^1^H-NMR and XRD measurements notified that π–π stacking and hydrogen bonding were the main driving forces for gelation. Upon pH variation, the morphology can be reversibly altered from nanofibers to vesicles and nanotubes. The 3D fibrous network with diameters from 50 to 200 nm was very stable under neutral and basic conditions, but transmitted into the sol state, consisting of vesicles and nanotubes (in the range of 50–300 nm), by adding puffer solution with pH = 4. This was explained by the interference of hydrogen-bonding implemented by amide groups. Subsequently, they incorporated curcumin, a chemopreventive drug, into the hydrophobic part of the hydrogel and released it by switching from gel-to-sol provoked by external stimuli such as pH change or the addition of Fe^3+^ ions. The –OH and carbonyl-groups of the coumarin moiety were able to coordinate with the metal ions, which resulted in the disassembly of the composite gel and the release of the drug [157].

Along with drug delivery systems, gene delivery agents also play an important role in medical science. They enable the study of interaction between nucleic acids (RNA and DNA) and synthetic polymers to elaborate new methods in genetic engineering and gene therapy. Gel electrophoresis is the commonly used method to detect these interactions and to estimate the best nucleic acid—polymer ratio. To visualize the electrophoresis results, fluorescence dyes have to be applied by (i) using intercalation agents, (ii) marking nucleic acid, or (iii) marking the synthetic polymer. Recently, Annenkov et al. have tagged the synthetic polymers poly(vinyl amine) (PVA) and polyethylenimine (PEI) with succinimidyl esters of 7-(diethylamino)coumarin-3-carboxylic acid (SECCA) to see unbound polymer and to use various nucleic acids during electrophoresis. The electrophoresis experiments exhibited movement of the polymers to the negative electrode due to positively charged polymers. By increasing the DNA content in the polymer–DNA mixture, the positive charges decreased and thereby also the movement to the negative electrode, which facilitated the study of interactions between polymer and nucleic acids [158].

### 5.4. Bioimaging

Magnet resonance imaging (MRI), computed tomography (CT), single-photon emission computed tomography (SPECT), and positron emission tomography (PET) are commonly used as noninvasive bioimaging modalities to diagnose and to treat diseases. As a next step, molecular imaging methods could be implemented to evolve the medical imaging sector [159]. Per definition, molecular imaging means “… in vivo characterization and measurement of biological processes at the cellular and molecular level” [160]; generally speaking, it is a method to control the distribution of molecular and cellular processes in biochemistry, biology, and diagnostic and therapeutic field. One approach of bioimaging is based on fluorescence imaging, which is a powerful tool for the visualization of molecules in living cells or tissues. It is highly sensitive, rapid, versatile, and requires only a low expressing cellular marker [161]. Due to the fluorescence properties of coumarin derivatives (see Section 4) and their biocompatibility, it is an interesting molecule for molecular imaging and there already exist some studies regarding the use of it in polymers for cell imaging applications.

One prospect is the incorporation of coumarin into dendrimers for bioimaging applications owing to their well-defined size, structure and shape, low toxicity, and immunogenicity and their wealth of numerous reactive functional groups on the surface [162]. Goonewardena and coworkers produced a fluorogenic dendrimer by copper(I)-catalyzed azide-alkyne cycloaddition reaction to follow cellular processes. In detail, they synthesized a dendrimeric reporter system consisting of polyamidoamine (PAMAM) and 3-azido-7-hydroxycoumarin to overcome the poor water solubility and background fluorescence of conventional small molecule reporters and to improve probe flexibility. By introducing an azido-functionality at the third position of coumarin, the fluorescence was quenched because of the electron-donating effect. However, based on the electron delocalization by the formation of the triazole ring during the reaction, the fluorescence of coumarin could be restored. The synthesized dendrimer reporter was used to observe the incorporation of 5-ethynyl-20-deoxyuridine (EdU) into DNA, which is a conventional molecular biology method to monitor cellular proliferation. Using a dendrimeric scaffold instead of small molecule reporters maintained fluorescent quenching in biological matrices, and metabolites could be profiled without a washing step, which improves assay performance [107].

Yeo et al. produced coumarin-containing dendrimers that formed conjugates with malaria antigens for malaria immunodiagnostic. The fluorescent intensity correlated with the antigen amount with high sensitivity [110]. The same group also investigated a method to detect influenza A viruses using the same coumarin-containing dendrimer in a fluorescent immunochromatographic strip test to improve the sensitivity of quantitative rapid diagnostic tests (RDT). The test relied on conjugation of coumarin-containing dendrimers with latex beads and antibodies to obtain fluorescent emission in broad spectral ranges. A sufficient assignment of the fluorescent emission of the beads was achieved by introducing a long-pass optical filter remote from the excitation wavelength. The dendrimers targeted the influenza A nucleoproteins and the resulting fluorescence intensities were used to distinguish between avian and human influenza A viruses. The newly investigated test performed 2.5-fold more sensitively than conventional dot blot immunoassays or RDTs and was able to prove four different avian influenza A subtypes to differ from other viral diseases and to enable quantification of the infection [111].

**Figure 31:**
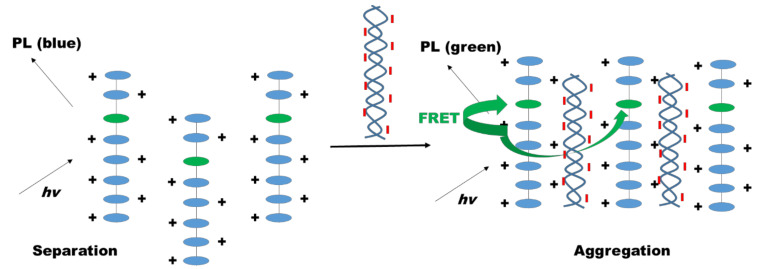
Schematic representation of the fluorescent resonance energy transfer (FRET) of the water-soluble electrolyte by addition of DNA. Adapted from [110].

Apart from dendrimers, coumarin-containing conjugated polyelectrolytes can also be used for cell imaging applications. A group from China revealed three new polyelectrolytes derived from coumarin, i.e., carbazole, fluorene, and phenylene derivatives by Suzuki coupling reaction for DNA sensors and effective fluorescent cell labeling agents (Figure 31). They synthesized different monomers bearing narrow bandgap coumarin-carbazole units or wide bandgap fluorene-phenylene units, which were copolymerized in different ratios and subsequently ionized. The cationic polymers formed energy donor-acceptor architectures, where the coumarin-carbazole unit acts as acceptor of fluorescent resonance energy transfer (FRET) from fluorene-phenylene (energy donor). By adding calf thymus DNA, an efficient FRET could be observed in terms of a fluorescent color change from blue to light green. Therefore, the water-soluble P1 electrolyte was used as fluorescent probe for the imaging of fibroblast cells of human adult skin, since blue and green fluorescences were monitored in the cytoplasm using different excitation wavelengths [108].

Moreover, organogels and self-assembled nanostructures are feasible materials for cell imaging applications. Recently, Lalitha and Nagarajan have investigated pyrene-coupled coumarin derivatives with varying hydrophobic units by aldol condensation to study their gelation behavior and self-aggregation properties in dependency on molecular structure and solvent affinity. In long-chain alcohols, the formation of a self-assembled molecular gel was observed, while in aqueous media nanoflakes were obtained. Π–π stacking in combination with hydrogen bonding between carbonyl groups of coumarin-coupled pyrene with hydroxyl groups of the solvent were suggested as the main driving forces for gelation and self-assembly process. The self-assembled nanostructures were successfully applied in the imaging process of fibroblast and PC3 prostate cancer cells [112].

## 6. Self-Healable Polymers with Coumarin as Reactive Moiety

**Figure 32:**
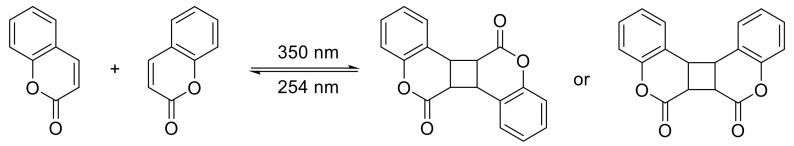
Reversible photodimerization of a coumarin ring.

In recent years, self-healing polymers have come to the forefront in a new class of smart materials with a powerful ability to automatically repair the damage inflicted on them without any external force [163,164]. Numerous promising applications of self-healable polymers can be found in the automotive, construction, and varnish and paint industries as well as electronics, aerospace, medicine, rubber production, and special-purpose materials [165,166]. Self-healing materials can generally be divided into two categories: extrinsic and intrinsic self-healing. Extrinsic self-healing materials are based on the pre-embedded healing agent, which is activated after cracking [164]. In contrast, intrinsic self-healing materials enable recovery without an additional healing agent due to the possibility of the formation of a covalent bond (e.g., Diels-Alder reaction or [2πs + 2πs] cycloaddition), radical-based systems, ionic interactions, metal–ligand interactions, supramolecular interactions, π–π interactions, or host–guest interactions [167,168,169]. The most important advantage of using coumarin moiety is its capability of undergoing a reversible photodimerization [170,171]. Photostimulated self-healing of polymers is quite attractive because the use of light is environmentally friendly and inexpensive and light is readily available [172]. The photodimerization of coumarin and its derivatives both in solution and in the solid-state by ultraviolet irradiation has been studied extensively [163,173]. As shown in Figure 32, the [2πs + 2πs] cycloaddition reaction to form a cyclobutane ring can take place in reversible photoinduced reactions of coumarin. Thus, reversible reactions of the formation of the ring are achieved by 350 nm radiation, whereas cleavage is achieved by the exposure to 254 nm radiation where the former coumarin moieties are formed [62,174,175].

**Figure 33:**
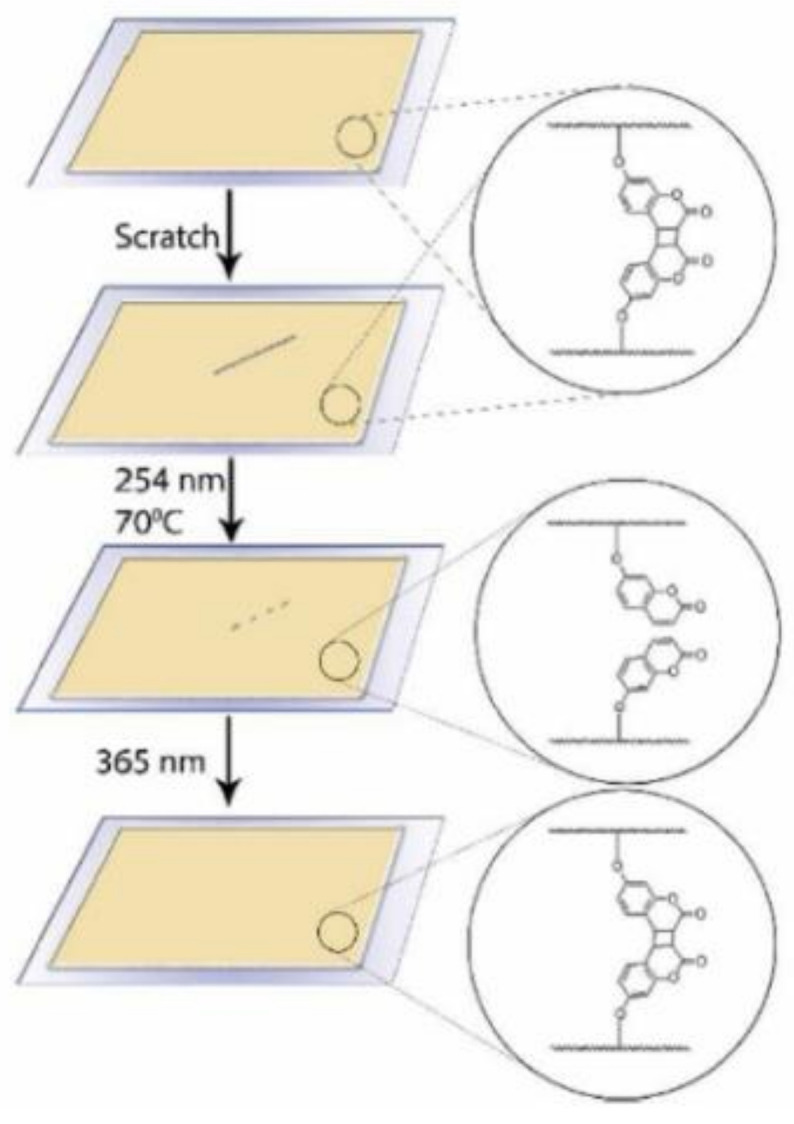
Schematic illustration of self-healing of polymer networks based on the reversible photodimerization of coumarin. Republished with permission of the Royal Society of Chemistry, from [179]; permission conveyed through Copyright Clearance Center, Inc.

The previously mentioned unique properties make coumarin a promising candidate as a stimuli-responsive unit in self-healing polymers. In the last few years, intensive research has been done on new polymers containing coumarin chromophores. Coumarin groups have been integrated into the backbone of various types of polymers such as polyethers [29], polyacrylates [62], polyesters [85], silicones [176], and polyurethanes [177,178]. The photo-induced repair of polymers with coumarin groups typically follow three steps after crack insertion (Figure 33). In the first step, the crack planes are exposed to deep UV-light (254 nm), which induces a cleavage of covalent cross-links corresponding to a regeneration of the original coumarin moieties. The network cleavage leads to a fluidification of the material, which can be enhanced by additional heating at the same time. Diffusion of the mobile and decross-linked polymer chains takes place leading to a physical healing of the damage zone. In a subsequent step, a chemical healing of the crack is obtained by illuminating the damage zone with longwave UV-light and the polymer network is recross-linked by the optically triggered dimerization of the coumarin groups upon UV irradiation with 365 nm [179].

In 2017, Saito and coworkers reported a convenient synthesis of a functional polymer, which consisted of acrylate monomers (butyl methyl acrylate (BMA), methyl acrylate (MA), hexyl methacrylate (HMA) and ethyl acrylate (EA)) and photoreversible coumarin moiety [179]. The hardness of the formed cross-linked polyacrylates was studied prior to and after healing, for the purpose of understanding mechanical properties. UV light was applied to repair the damaged films. In some cases, heating was combined with UV light to induce sufficient mobility of the linear polymer chains to fill the damaged part of the films. The disappearance of scratches was observed by the naked eye. Further, in order to investigate the self-healing properties, glass transition temperature (*T*_g_) was tested for both virgin and irradiated samples. The obtained results are summarized in Table 2.

The *T*_g_ values of samples irradiated with 254 nm UV light were lower that the virgin samples, which can be explained by the decrease in cross-link density due to cleavage of the coumarin dimers.

**Figure 34:**
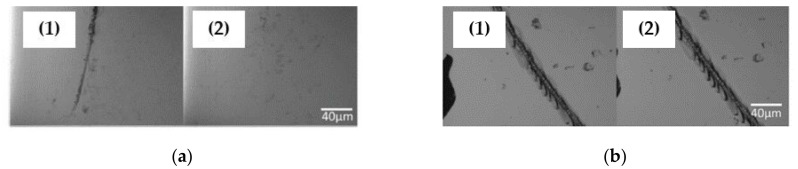
Optical images of damaged and repaired EA copolymers containing 3% of 7-methacryloyloxy coumarin as photoreactive pendant groups. (**a**) Healing an inserted crack (1) using light only (2) and (**b**) healing an inserted crack (1) by heating only (2). Republished with permission of the Royal Society of Chemistry, from [179]; permission conveyed through Copyright Clearance Center, Inc.

However, subsequent UV exposure with 365 nm leads to a recross-linking of the polymer chains, which is attributed to an increase in the *T*_g_. The ability of the networks to heal-inserted cracks is shown in Figure 34a.

The visual disappearance of the scratches can be noticed, while other regions were repaired to about 80% of their virgin state. Control experiments (Figure 34b) clearly indicate that healing of the surface is only possible due to the photoreversible reaction of the coumarin pendant groups. Although thermal energy encourages molecular mobility, by applying just a heat would not lead to healing.

**Figure 35:**
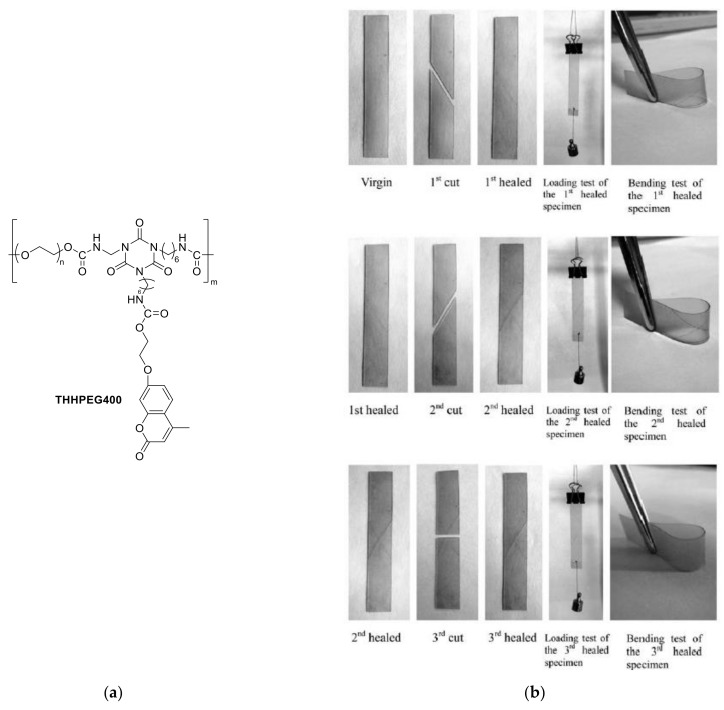
(**a**) The chemical structure of THHPEG400. (*b*) Macroscopic repairing of crossed ruptures of cross-linked THHPEG 40 with the UV light. Republished with permission of the Royal Society of Chemistry, from [69]; permission conveyed through Copyright Clearance Center, Inc.

For several years, a great effort has been devoted to the study of properties of coumarin groups in polyurethane [178]. Ling and coworkers developed several strategies to incorporate coumarin into polyurethanes for the purpose of self-healing ability. In 2011, they introduced coumarin side groups into the main chains of the solid polyurethane. They synthetized a trifunctional homopolymer of hexamethylene diisocyanate with polyethylene glycol as the skeleton and 7(hydroxyethoxy)-4-methylcoumarin as the pendant group [69]. The structure of THHPEG400 is shown in Figure 35a. A reversible photodimerization of the pendant coumarin groups takes place either by ultraviolet irradiation or under direct sunlight. To evaluate the healing efficiency of the photoresponsive polyurethane, a virgin specimen was cut by a razor for three times. The second cut was made across the first one that was successfully healed and the third cut was made horizontally across the intersection of the former two cuts (Figure 35b). Self-healing was performed by irradiating the fractured surfaces of the broken specimen with 254 nm UV light for 1 min, and then irradiating the sample with 350 nm for 90 min.

## 7. Shape-Memory Polymers with Coumarin as Reactive Moiety

Shape-memory polymers (SMPs) are a class of polymeric smart materials that attract increased interest due to their remarkable property to return from a deformed state to an original shape [180]. SMPs change their shape when a particular stimulus is applied such as light [181], heat [182], electric field [183], magnetic field [184], sonic field [185], solvent ions [186], pH value [187], specific antigen–antibody interactions [188] and others. Due to this unique feature, they are successfully applied as smart medical devices [189,190], thermal sensors and actuators [191,192,193], and smart textiles [194]. In 2005, Lendlein et al. have reported an approach of introducing photosensitive functional groups into a polymer matrix to prepare optically triggered SMPs [195]. Due to a wide range of availability of photoreactive molecules and good irradiation selectivity, photochemical activation is probably one of the most used stimuli in this field. Coumarin chromophores have been inserted into the polymer system such as polyesters [196,197,198,199], polyvinyl alcohol (PVA) [200], polyurethane [201,202], and poly(4-vinyl pyridine) [203].

In 2009, Nagata and Yamamoto have reported the synthesis of high-molecular-weight photosensitive biodegradable polyesters with a pendant coumarin moiety. The synthesis was carried out by polycondensation solution from 7-(3,5-dicarboxyphenyl) carbonylmethoxycoumarin (ICM) dichloride and polycaprolactone (PCL) diols (*M*_W_ = 1250, 3000, and 10,000 g/mol). The shape-memory behavior was studied by conducting a cyclic thermomechanical experiment to determine strain fixity ratio (*R_f_*) and strain recovery ratio (*R_r_*). The cross-linked ICM/PCL-3000 and -10,000 films have shown excellent properties in which both *R_f_* and *R_r_* were 88–100% for a strain of 100–500% [199].

**Figure 36:**
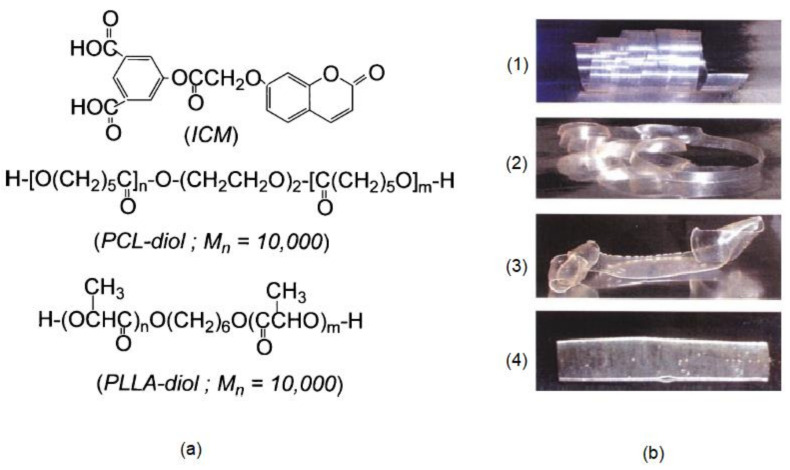
(**a**) Chemical structures of used compounds. (**b**) The image of shape-memory effect of photocross-linked 7-(3,5-dicarboxyphenyl) carbonylmethoxycoumarin (ICM)/polycaprolactone (PCL)-10,000 for transition from temporary (1) to permanent shape (4) at 60 °C. Adapted from [199], with permission from John Wiley and Sons.

One year later, the same authors have investigated the synthesis and shape-memory properties of a series of photocurable block copolymers of *ε*-caprolactone and *L*-lactide by polycondensation of poly(*L*-lactide) PLLA diol (*M*_W_ = 10,000 g/mol), PCL diol (*M*_W_ = 10,000 g/mol), and a chain extender bearing a coumarin group (Figure 36). The photosensitive chain extender was synthesized from 7-carboxymethoxycoumarin and 5-hydroxyisophthalic acid. ICM/PCL and block copolymers with higher PCL content (≥75 mol.%) showed good to excellent shape-memory properties. *R_f_* and *R_r_* values of photocured copolymers for the third cycle were 97–100% and 76–100%, respectively, at a recovery temperature of 60 °C for different tensile strains of 100–500% [198].

**Figure 37:**
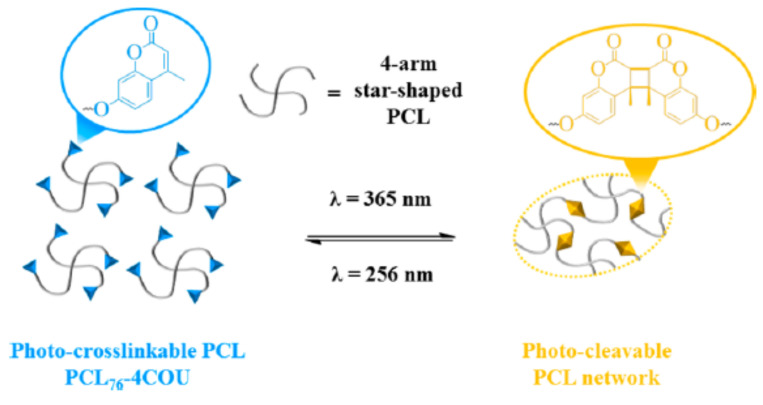
Schematic illustration of coumarin-based photocross-linkable star-shaped PCL for light-controlled design of network and shape reconfiguration. Reprinted with permission from [196]. Copyright (2019) American Chemical Society.

Excellent shape-memory properties have been achieved for PCL_76_-4COU by Jérôme and coworkers in 2018. A 4-arm star-shaped PCL was functionalized by 4-methycoumarin by UV irradiation at 365 nm (Figure 37).

Swelling experiments demonstrated that an almost quantitative conversion of coumarin can be reached after 360 min of UV irradiation, while Raman spectroscopy analysis showed that by addition of benzophenone before UV curing full conversion can be achieved after 30 min. Shape-memory properties of these well-defined coumarin-based materials can be easily controlled due to a homemade mold design. PCL-based SMP materials have shown high fixity rate, and recovery has been achieved within 5 min irradiation [196].

In 2014, Zhang et al. have reported the insertion of coumarin into polyvinyl alcohol (PVA). The photosensitive polymer (PVA-coumarin) was prepared by esterifying the hydroxyl groups of PVA with 7-carboxymethoxycoumarin with different degrees of substitution (DS). The material was photocross-linked after UV light irradiation at 360 nm. The sample has shown shape-memory properties only at a high degree of substitution leading to higher cross-linked networks. Further, thicker samples suffered from lower cross-link density, which was overcome by longer irradiation time [200].

**Figure 38:**
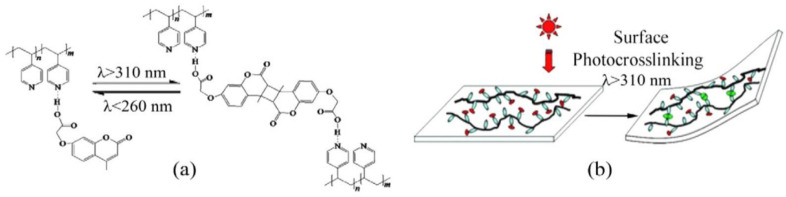
(**a**) Reversible photocross-linking of P4VP partially complexed with 7-(carboxymethoxy)-4-methylcoumarin through hydrogen bonding. (**b**) Scheme of the mechanism underlying the photoinduced bending of the film. Republished with permission of the Royal Society of Chemistry, from [203]; permission conveyed through Copyright Clearance Center, Inc.

In 2008, Zhao and coworkers have reported the reversible photodimerization of poly(4-vinyl pyridine) (P4VP) partially complexed with 7-(carboxymethoxy)-4-methylcoumarin through hydrogen bonding between the pyridyl and carboxylic acid groups (Figure 38a).

As is shown in Figure 38b, the photodimerization on the surface proceeds upon irradiation with UV light using wavelengths above 310 nm, which results in large bending of the sample. The degree of the photoactive bending deformation could be compared with that of photoactive liquid crystal elastomer obtained from azobenzene-based polymers [203].

**Figure 39:**
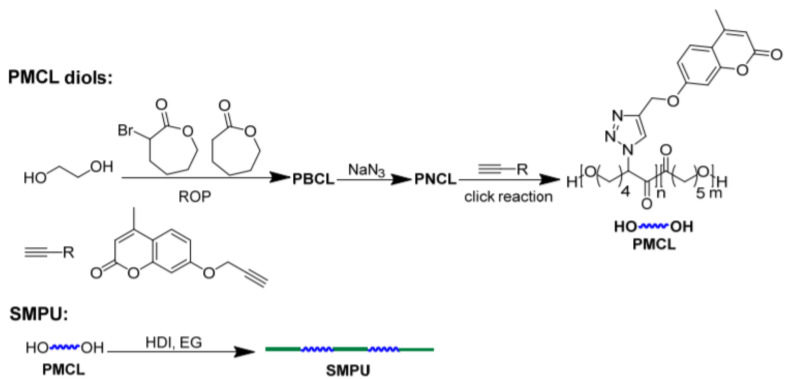
Synthetic route of shape-memory polyurethanes (SMPU) containing coumarin moieties. Reprinted from [202], Copyright (2019), with permission from Elsevier.

In 2019, Chen has reported two papers about coumarin insertion into a polyurethane network by exploiting “click” chemistry. In the first paper, the authors described the synthesis of triple-shape-memory polyurethanes (SMPUs) based on photoreversible coumarin units linked with poly(ε-caprolactone) (PMCL) soft segment by ring-opening polymerization (Figure 39) [202].

The triple-shape-memory effect versus UV irradiation time was studied by cyclic mechanical experiments. The results are summarized in Table 3, referring to the total shape fixity ratio (*R_f_*_(A to B)_), shape recovery ratio *R_r_*_(B to A_), and the ratios of first and second recovery steps.

**Figure 40:**
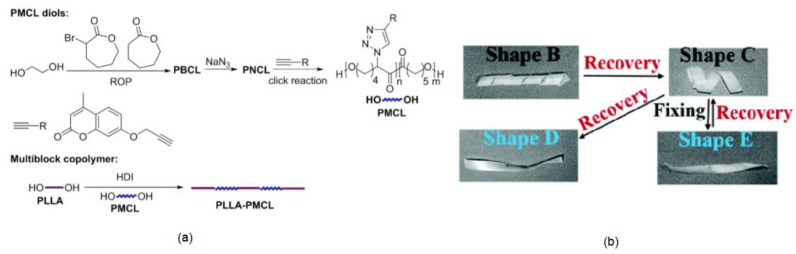
(**a**) Synthetic route of multiblock polymers PLLA-PMCL1. (**b**) Visual demonstration of the triple-shape-memory performance of PLLA-PMCL. Republished with permission of the Royal Society of Chemistry, from [201]; permission conveyed through Copyright Clearance Centre, Inc.

The second paper of Chen et al. describes triple-shape-memory effect (triple-SME) in photoresponsive coumarin-containing PMCL as soft segments and poly(L-lactide) (PLLA) as hard segments (Figure 40a) [201].

Figure 40b shows a series of photographs visualizing a one-step deformation programming procedure as well as staged recoveries of shapes B and C, which demonstrate the triple-SME of PLLA-PMCL1. Moreover, a one-way shape-memory programming procedure and recovery of shape E is provided to illustrate the thermally sensitive SME using shape C as a permanent shape. Triple-shape-memory properties of PLLA-PMCL copolymers for various time irradiation and light intensities are summarized in Table 4.

In both cases, the coumarin was introduced in the polymer matrix by a “click reaction.” To achieve the shape-memory effect, it was important to control the molecular weight of the soft segment as well as the composition of polyurethane to obtain crystallization of both soft and hard segment. In summary, the authors found a new method to achieve a UV/heat dual-responsive triple-shape-memory effect in photoresponsive polyurethanes. The recovery ratios of temporal shapes (Figure 40b, shapes B and C) could be easily adjusted by only tuning the time, photocross-linking intensity, and heating of the sample. The temporary shapes of these copolymers were found to be very stable and used as a complex permanent shape for thermally sensitive SMPs. With these beneficial properties, the described polyurethanes can be used in a wide range of applications such as soft robots, medicine, and textile.

## 8. Polymers with Coumarin in Soft Robotics Applications

**Figure 41:**
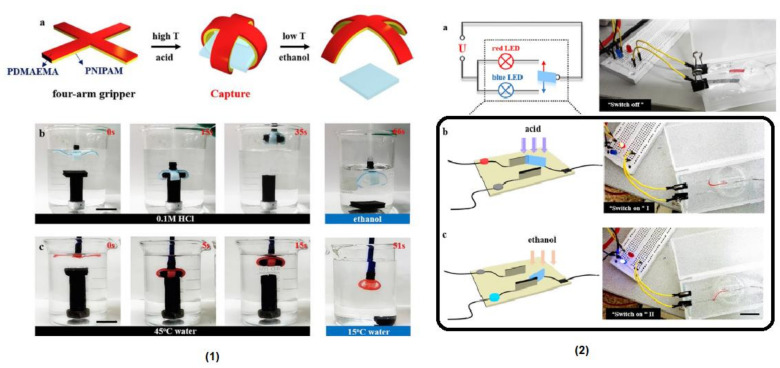
(**1**) Scheme (a) and photographs of bilayer hydrogel as a four-arm gripper to capture the weight in response to pH (b) and temperature (c). (**2**) Scheme and photographs of the bilayer-type hydrogel as the bidirectional circuit switches: the disconnected state (a) and the connected state that turns on the red (b) or blue (c) light emitting diodes (LEDs). Reprinted from [218], Copyright (2018), with permission from Elsevier.

Soft active hydrogels are three-dimensional networks that have become very popular due to their excellent properties such as biocompatibility, flexibility, softness, and water content [204]. Under the influence of external stimuli, such as temperature [205,206], pH [207,208,209], chemicals [210], or light [211,212], a hydrogel undergoes defined deformation and shape changes. Taking advantage of that properties, they can be used in various applications, such as drug delivery systems [213], sensors, and actuators for soft robots and soft machines [214,215,216]. In 2017, Wei and coworkers have fabricated a bilayer-type fluorescent hydrogel soft actuator that responds to temperature and the pH value. It was based on two layers of hydrogels with different swelling rates. The first layer was a positive layer, which responded to specific stimuli by swelling on shrinkage, while the second layer was a negative layer that had no response to the same stimuli [217]. The bilayer hydrogel by Wei et al. contained poly(*N*-isopropylacrylamide) (PNIPAM) and poly(2-(dimethylamino) ethyl methacrylate) (PDMAEMA), which separately served as temperature- and pH-sensitive layers. Poly(ethylene oxide) diacrylate was present in both layers and served as a cross-linker. The photo-sensitive polymerizable coumarin-based monomer was inserted into the hydrogel network to add fluorescence. Further experiments have shown fast-responding behavior in terms of response to both temperature and pH activation. As is shown in Figure 41, a four-arm gripper and a circuit switch were designed to prove the stimuli-responsive actuations. It resulted in accurate capture of the rubber bulk and bidirectional alarm to turn on the LEDs in response to the varying environments, which leads to potential applications in the fields such as soft actuators, ionic circuity, environmental sensors, and biomimetic devices.

According to the authors’ knowledge, there are only a few studies where a positive layer is capable of responding to only one or two stimuli [218]. Johnson in 2020 was the first who reported a polymer gel comprising poly(ethylene glycol) star polymer linked by Cu_24_L_24_ metal-organic cages/polyhedra (MOCs) with coumarin ligands. The “polyMOC” materials can be reversibly switched between Cu^II^, Cu^I^, and Cu^0^ state in the presence of UV light, a photosensitizer, and a hydrogen donor.

**Figure 42:**
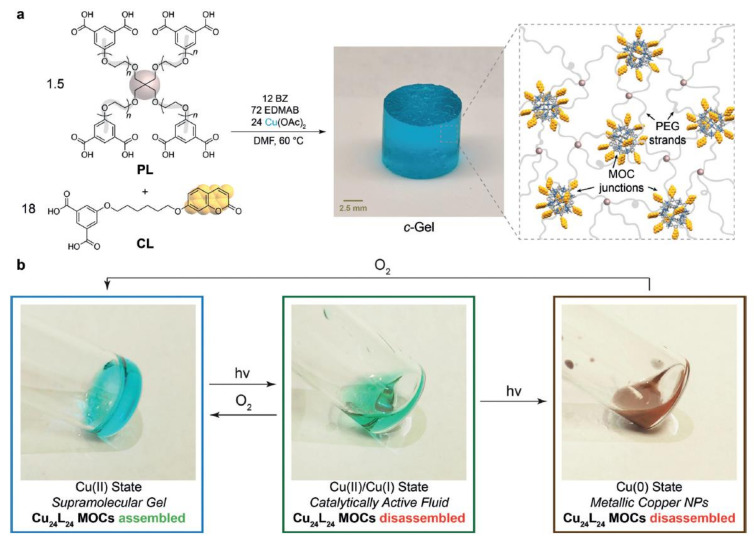
(**a**) Schematic illustration of Cu_24_L_24_-based metal-organic cages/polyhedra (MOCs) with coumarin-functionalized junctions. (**b**) Reversible switching of c-Gel between redox states (Cu^II^, Cu^I^, and Cu^0^) with corresponding gel–sol transitions and differences in catalytic activity. Adapted from [219], with permission from John Wiley and Sons.

The instability of the MOC junctions in the Cu^I^ and Cu^0^ state is resulting in network disassembly whilst the formed Cu^I^/Cu^0^ solutions are stable until reoxidation to Cu^II^ and supramolecular gelation. The driving force of this reversible disassembly of the polyMOC network is in situ generated copper-catalyzed azide-alkyne cycloaddition (CuAAC), which occurs in a fixed covalent second network, resulting in interpenetrating supramolecular and covalent networks. The synthesis of “polyMOC” was carried out by mixing *m*-BDC-functionalized PEG star polymer PL and coumarin-functionalized *m*-BDC (**CL**) with Cu(OAc)_2,_ dimethylformamide (DMF) as a solvent, benzophenone (BZ) as a photosensitizer, and ethyl-4-(dimethylamino)benzoate (EDMAB), which acts as H-atom donor. polyMOC c-Gel, which is composed of Cu24L24 junctions decorated with coumarin chromophores (CL) and polymers strands (PL), was then obtained by annealing (Figure 42a).

As is shown in Figure 42b, with reversible switching of *c*-Gel between three stable redox states: Cu^II^, Cu^I^, and Cu^0^, a transitioning between sol–gel transitions and catalysis within the polymer network was demonstrated [219]. The authors have claimed that the resistance of the Cu^0^ regions of the described material was approximately 1000-fold lower compared to nonirradiated regions. Based on previously published work, further engineering, sintering, and optimization of the switchability of *c*-Gel could be a key for opening new avenues for the fabrication of integrated soft-material circuits that could be applied in sensing or soft robotics [220,221].

## 9. Polymers with Coumarin in Tissue Engineering Applications

Tissue engineering is a multidisciplinary field of biomedicine based on replacing or curing damaged organs. Apart from the scientific community, tissue engineering is undoubted a hot topic in practical medicine. A medical study has shown that more than 8 million surgical treatments are performed with the aim of replacing damaged organs with costs of more than $400 billion per year [222,223]. Natural and synthetic polymers, as well as, ceramics have been used as the chemical composition of scaffold or hydrogels that are playing an important role in providing mechanical support during in vivo implantation [224]. Polymeric materials possess a wide range of different properties such as mechanical properties, biodegradation, small pore size, as well as high porosity and surface-to-volume ratio that can be used in multiple applications for engineering and regenerating hard and soft tissues [225,226]. Compared to synthetic polymers, natural polymers, also known as biopolymers, are obtained from renewable resources with great potential to elude immunological reactions or chronic inflammation toxicity. For these reasons, they are widely used for drug delivery and designing therapeutic systems as well as bioengineer functional tissues. On the other hand, properties of synthetic polymers such as mechanical strength, degradation rate, elastic modulus, and tensile strength can be modified for the specific application [227,228]. In tissue engineering, coumarin is often chosen as a photocross-linkable group because it exhibits photosensitivity, biodegradability, and photo reversibility [229].

**Figure 43:**
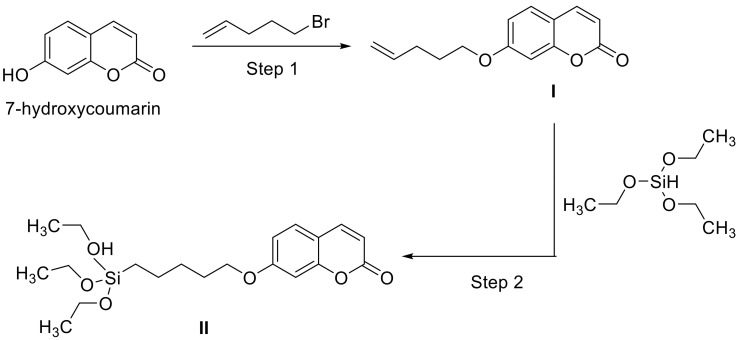
Synthesis of coumarin derivatives: **I** and **II**.

Since their discovery by Hench et al. in 1971, bioactive glasses (BG) are well-known materials used in medical applications. The unique properties such as nontoxicity, without inducing inflammation as well as immune response, make BGs easily applicable in bioactive coatings of metallic implants in tissue engineering, clinical tissue regeneration, and tissue engineering [230,231,232,233]. In 2004, mesoporous bioactive glasses (MBG) have been developed by Yan et al. [234]. These new class of biomaterials has a higher specific surface area and pore volume compared to BG, which may open up new opportunities in tissue engineering. In 2010, Shyu and coworkers reported grafting of a photoresponsive coumarin derivative onto MBG solids to develop photocontrolled molecular gates. The first step includes the synthesis of 7-pentenylloxycoumarin (**I**) starting from 7-hydroxycoumarin. In the second step, the reaction between the obtained product **I** with triethoxysilane resulted in the 7-((3-triethoxysilyl)pentyloxy)coumarin (**II**) (Figure 43).

**Figure 44:**
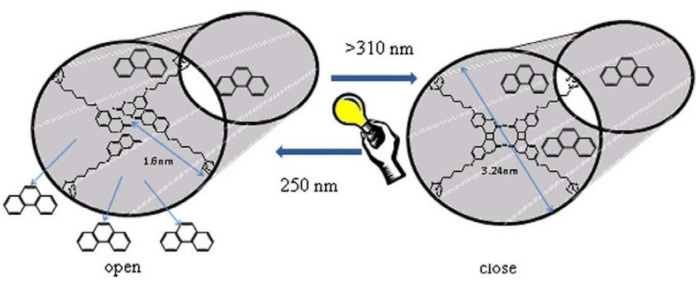
The concept of the photocontrolled “open-close” mechanism. Reprinted from [42], Copyright (2010), with permission from Elsevier.

In the final step of modification, the synthesized MBG was mixed with the obtained product **II** yielding coumarin-modified MBG. The photocontrolled “open-close” mechanism is based on the dimerization and cleavage of the photoactive coumarin moiety, which is controlled by different wavelengths of UV irradiation. As is shown in Figure 44, photocleavage of coumarin dimers is induced by irradiation at 250 nm, which led to the pores’ opening. Further, the photodimerization is induced by longer wavelength (>310 nm), which facilitates a closing of the pores. The described photocontrolled mechanism is reversible, and the guest molecule can be released or entrapped depending on UV wavelength irradiation [42].

Hydrogels are attractive materials for different applications in the field of tissue engineering as important forms of bioscaffolds [235]. Several groups published research on the photocross-linked hydrogels with the pendant coumarin group [175,236,237,238].

**Figure 45:**
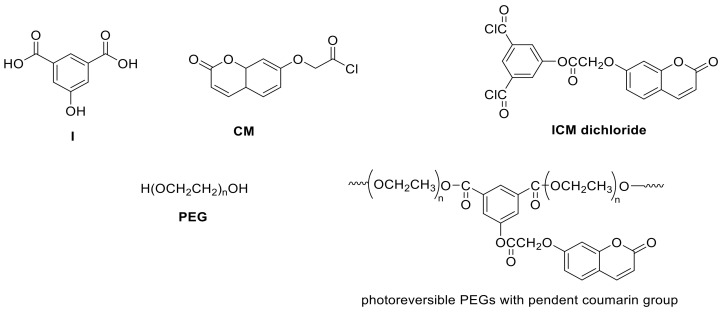
Components used in the synthesis of photoreversible poly(ethylene glycol)s with pendant coumarin group.

Yamamoto et al. have used the photoreversible dimerization of 7-chlorocarbonylmethoxycoumarin (CM) for the development of photoreversible poly(ethylene glycol) derivatives (PEG)s (Figure 44). Due to their properties such as swellability in water, hydrophilicity, and lack of toxicity, PEG hydrogels have shown good potential in tissue engineering and drug delivery carriers. The synthetic route of the photosensitive chain extender (ICM) includes condensation of 5-hydroxyisophthalic acid (I) and CM. In the next step, obtained ICM dichloride was polycondensed with (PEG)s of different molecular weights (MW = 1000, 3000, and 8300) in the absence of a photoinitiator (Figure 45).

The PEG hydrogels were formed in a very short time under UV exposure with wavelengths >280 nm and were swollen in water to test the properties. Within 5 min, the gel contents increased rapidly and attained over 90% for ICME3000 and 80% for ICME8300. It is possible to control the degree of swelling by the irradiation time as well as MWs of PEG [175].

Taking advantage of hyaluronic acid (HA) properties such as high water content, good biocompatibility, capacity to degrade into safe products, and ability to imitate the extracellular matrix (ECM) environment [239], Elvassore et al. have developed an in situ preparation of hydrogels based on HA. In the field of cross-linking chemistry, previously published papers were carried out in the presence of a coupling agent or a photo-/radical initiator which can generate exothermic reactions and cause a negative influence on surrounding tissues [240,241]. This problem was solved by Borsato and coworkers by involving of coumarin moieties in the formation of cross-linked HA hydrogels [242]. In 2018, Elvassore et al. described a noncytotoxic and aqueous/safe photocross-linking process that takes place under near-UV light irradiation at 365 nm resulting in two new covalent bonds for each site for a time ranging from 3 to 5 min, without formation of by-products.

**Figure 46:**
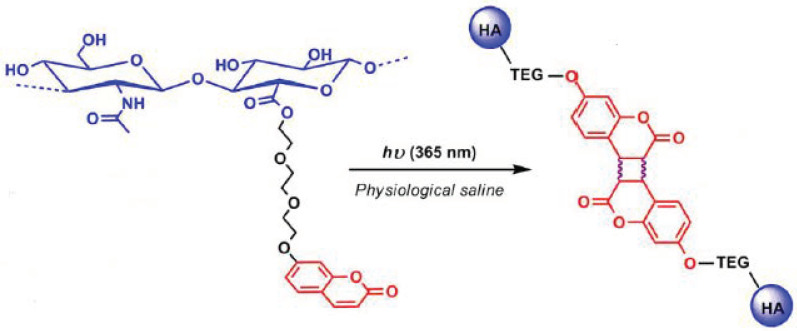
Photocross-linking of hyaluronic acid (HA)-triethylene glycol (TEG) coumarin. Reprinted from [236], Copyright (2019), with permission from Elsevier.

As is shown in Figure 46, triethylene glycol (TEG) plays an important role as a linker between the HA and coumarin moiety (7-hydroxy coumarin) to achieve hydrophilicity and encourage the matching between coumarin moieties in aqueous solutions due to improved conformational freedom. In summary, the hydrogel obtained with this technique is radical-free, noncytotoxic, biodegradable, as well as biocompatible. These unique properties make the described hydrogel a good candidate for biomedical applications, for instance, in tissue regeneration applications acting as efficient scaffolds [236].

**Figure 47:**
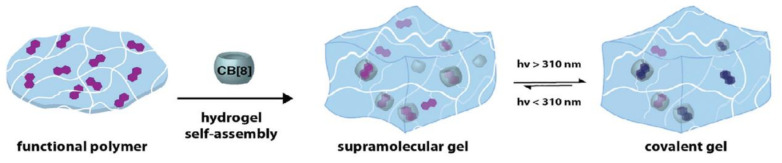
The concept for photo-switchable coumarin-functionalized biopolymers. Republished with permission of the Royal Society of Chemistry, from [237]; permission conveyed through Copyright Clearance Center, Inc.

Particular interest was also given to photo-switchable supramolecular hydrogels. The benefit of these materials is the ability to modulate stiffness with light resulting in photo-tunable hydrogels. In 2018, Scherman and coworkers reported supramolecular coumarin-functionalized hydrogels that can switch from a supramolecular gel into a covalent state. A number of studies have found coumarin as a promising guest for macrocycles such as cucurbiturlis [243,244,245]. By following this idea, the authors developed a strategy for the functionalization of hyaluronic acid (HA) and hydroxyethyl cellulose (HEC) with coumarin in the presence of cucurbit[8]uril (CB[8]) based on host–guest chemistry. The supramolecular gel is made from HA or HEC with a pendant coumarin group mixed together with CB[8] in solution. Coumarin undergoes a [2πs + 2πs] cycloaddition upon UV exposure at >310 nm resulting in the formation of covalent cross-links between the polymer chains (Figure 47).

Further analysis of the photoreversibility of these functional materials has shown that hydroxyethyl cellulose-coumarin (HEC-COU/CB[8])-based gels could be photocured and dimerized from a supramolecular gel into a covalent state, but could not be photoreversed back. While hyaluronic acid-coumarin (HA-COU/CB[8]) has shown photoreversible properties for at least two cycles as well as shear-reversible properties. However, this system could not be used in applications where the required irradiation is below 310 nm [237].

**Figure 48:**
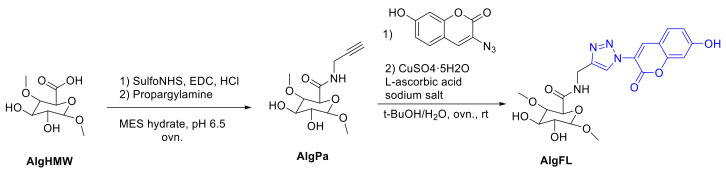
Synthetic route of fluorescent coumarin-grafted alginate.

In 2020, the Barrias′ group has reported the synthesis of coumarin-grafted blue-emitting fluorescent alginate (AlgFL), which can form mechanically stable hydrogels by ionic cross-linking. Previously reported studies on the fluorescence labeling were carried out in acidic environment using toxic reducing agents such as sodium borohydride or sodium cyanoborohydride [246]. Clinical applications should be based on the green synthesis where an acidic environment and usage of toxic reducing agents are avoided. AlgFL was obtained through carbodiimide chemistry in a click reaction of the 3-azido-7-hydroxycoumarin and the alginate using CuSO_4_·5H_2_O as a catalytic amount of Cu(II) source, under mild conditions in aqueous media (Figure 48).

The authors monitored fluorescent properties of the developed biomaterial, both in solution and hydrogel states. The polymer has shown a good capacity to fluorescence for long time periods, which makes this polymer a good candidate in biomedical assays where tracking of the material is required [238].

## 10. Conclusions

In summary, the introduction of coumarin chromophores into polymer-based materials offers a versatile and elegant approach to endow polymers with unique properties for a very wide range of applications. With the summarized applications in this review, we have shown that the development of polymers containing coumarin moieties has made immense progress over the past decade. Excellent and usable photophysical and photochemical properties of coumarin derivatives such as tuneable absorbance and fluorescence as well as photoreversible dimerization pave the way towards functional materials for electronics, biomedicine, energy harvesting, or soft robotics. Taken together, advanced properties, which have been highlighted in this review, are expected to continue to expand. In particular, natural coumarin derivatives have a high potential in future applications meeting on the one hand side the steadily increasing demand for materials derived from renewable resources and on the other hand providing a way to improve lifetime of polymer-based products by optically triggered self-healing concepts.

## Figures and Tables

**Table 1:** Thermal properties of the three different benzoxazine resins [141].

Resin	Melting Temperature (°C)	Polymerization Temperature (°C)	
		Onset	Max
PH-a	54	255	261
U-a	147	215	220
MU-a	153	229	232

**Table 2:** Glass transition temperature (*T*_g_) of virgin and irradiated acrylate copolymers containing 3% 7-methacryloyloxy coumarin as photoreactive pendant groups.

Polymers	*T*_g_ (°C)		
	Virgin Sample	Irradiation with 254 nm	Irradiation with 365 nm
**BMA**	70	52	67
**MA**	65	50	62
**HMA**	46	43	44
**EA**	–	32	35

**Table 3:** Triple-shape-memory properties of shape-memory polyurethanes (SMPU) containing coumarin moieties.

Sample	Photocross-Linking Conditions		*R_f_*_(0 to 1)_ (%) ^a^	*R_f_*_(1 to 0)_ (%) ^b^	∆*Ɛ_rel_*_(1 to 2)_ (%) ^c^	Δ*Ɛ_rel_*_(2 to 0)_ (%) ^d^
	Time (min)	Light Intensity (mW/cm^2^)				
SMPU	5	34.0	99	90.1	85.1	14.9
SMPU	10	34.0	99	92.1	79.1	20.9
SMPU	15	34.0	99	91	77.6	22.4

^a^ Fixed ratio of temporary shape 1. ^b^ Recovery ratio from shape 1 to original shape. ^c^ Ratio of thermal-responsive recovery step. ^d^ Ratio of photoresponsive recovery step.

**Table 4:** Triple-shape-memory properties of poly(L-lactide) (PLLA)-poly(ε-caprolactone) (PMCL) copolymer.

Entry	Sample	Photocross-linking Conditions		*R_f_*_(A to B)_ (%)	*R_r_*_(B to A)_ (%)	∆*Ɛrel*_(B to C)_ (%)	Δ*Ɛrel*_(C to A)_ (%)
		Time (min)	Light Intensity (mW/cm^2^)				
1	PLLA-PMCL1	1	27.8	99.0 ± 1.0	80.7 ± 1.2	86.2 ± 1.2	13.8 ± 1.2
2	PLLA-PMCL2	1	27.8	99.0 ± 1.0	72.2 ± 1.3	88.1 ± 1.3	11.9 ± 1.3
3	PLLA-PMCL2	0.5	27.8	99.0 ± 1.0	85.9 ± 1.2	88.0 ± 1.2	12.0 ± 1.2
4	PLLA-PMCL2	8	7.0	99.0 ± 1.0	89.4 ± 1.2	67.8 ± 1.2	21.5 ± 1.2
5	PLLA-PMCL1	10	34.0	99.0 ± 1.0	67.9 ± 1.3	47.5 ± 1.3	52.5 ± 1.3

^a^ Staged recovery conditions: (i) heating at 55 °C for 15 min and (ii) heating at 55 °C plus irradiation at *λ* = 254 nm for 2 h.

## Data Availability

Data is contained within the article or supplementary material.
